# Targeting PKCι-PAK1 signaling pathways in EGFR and KRAS mutant adenocarcinoma and lung squamous cell carcinoma

**DOI:** 10.1186/s12964-019-0446-z

**Published:** 2019-10-28

**Authors:** Masaoki Ito, Carles Codony-Servat, Jordi Codony-Servat, David Lligé, Imane Chaib, Xiaoyan Sun, Jing Miao, Rongwei Sun, Xueting Cai, Alberto Verlicchi, Morihito Okada, Miguel Angel Molina-Vila, Niki Karachaliou, Peng Cao, Rafael Rosell

**Affiliations:** 1 Pangaea Oncology, Laboratory of Molecular Biology, Quiron-Dexeus University Institute, Barcelona, Spain; 2 Laboratory of Cellular and Molecular Biology, Institute for Health Science Research Germans Trias i Pujol (IGTP), Badalona, Spain; 3 Department of Surgical Oncology, Research Institute for Radiation Biology and Medicine, Hiroshima University, Hiroshima, Japan; 4 IDIBELL/CReST/Translational Research Laboratori L’Hospitalet de Llobregat, Barcelona, Spain; 5 Hospital of Integrated Traditional Chinese and Western Medicine, Nanjing University of Chinese Medicine, Nanjing, China; 6 Laboratory of Cellular and Molecular Biology, Jiangsu Province Academy of Traditional Chinese Medicine, Nanjing, China; 7 Collaborative Innovation Center for Cancer Medicine, Nanjing Medical University, Nanjing, China; 8 Istituto Scientifico Romagnolo per lo Studio e la Cura dei Tumori (IRST) IRCCS, Meldola, Italy; 9 Institute of Oncology Rosell (IOR), Quiron-Dexeus University Institute, Barcelona, Spain; 10 Institut d’Investigació en Ciències de la Salut Germans Trias i Pujol Campus Can Ruti (Edifici Muntanya), Ctra. de Can Ruti, Cami de les Escoles s/n, Badalona, 08916 Barcelona, Spain

## Abstract

**Introduction:**

p21-activated kinase 1 (PAK1) stimulates growth and metastasis in non-small cell lung cancer (NSCLC). Protein kinase C iota (PKCι) is an enzyme highly expressed in NSCLC, regulating PAK1 signaling. In the present study we explored whether the PKCι-PAK1 signaling pathway approach can be an efficient target in different types of NSCLC cell and mouse models.

**Methods:**

The effect of IPA-3 (PAK1 inhibitor) plus auranofin (PKCι inhibitor) combination was evaluated by cell viability assay, colony formation and western blotting assay, using three types of NSCLC cell lines: EGFR or KRAS mutant adenocarcinoma and squamous cell carcinoma with PAK1 amplification. In addition, for clinical availability, screening for new PAK1 inhibitors was carried out and the compound OTSSP167 was evaluated in combination with auranofin in cell and mice models.

**Results:**

The combination of IPA-3 or OTSSP167 plus auranofin showed high synergism for inhibiting cell viability and colony formation in three cell lines. Mechanistic characterization revealed that this drug combination abrogated expression and activation of membrane receptors and downstream signaling proteins crucial in lung cancer: EGFR, MET, PAK1, PKCι, ERK1/2, AKT, YAP1 and mTOR. A nude mouse xenograft assay demonstrated that this drug combination strongly suppressed tumor volume compared with single drug treatment.

**Conclusions:**

Combination of IPA-3 or OTSSP167 and auranofin was highly synergistic in EGFR or KRAS mutant adenocarcinoma and squamous cell carcinoma cell lines and decreased tumor volume in mice models. It is of interest to further test the targeting of PKCι-PAK1 signaling pathways in EGFR mutant, KRAS mutant and squamous NSCLC patients.

## Background

Non-small cell lung cancer (NSCLC) is the leading cause of cancer related deaths and comprises several histological subtypes: lung adenocarcinoma (LUAD), squamous cell carcinoma (SCC) and large cell carcinoma. Despite the identification of targeted druggable driver mutations and rearrangements, most cases have poor survival [1, 2]. Recently, pembrolizumab plus chemotherapy have provided benefit in a fraction of patients, regardless of the level of PD-L1 expression [3–5]. However, the effect of immunotherapy in patients with EGFR mutations is rather limited [6]. A meta-analysis indicated that immuno-checkpoint inhibitors as second line treatment do not improve overall survival in comparison with docetaxel treatment in EGFR-mutant patients [7]. We focus our research on the identification of recurrent pathways occurring in subclasses of NSCLC, including LUADs driven by KRAS or EGFR mutations, and SCC. This stems from the fact that atypical protein kinase Cι binding to Par6 is associated with the epithelial cell transforming sequence 2 (Ect2), a guanine nucleotide exchange factor that activates Rac1 in downstream PAK1, MEK1/2-ERK1/2 signaling, regulating tumor growth in NSCLC [8–10]. PKCι is reported to be amplified in 20.2–36.5% of NSCLC patients, especially in SCC patients [8, 11]. PKCι mRNA is overexpressed in LUAD and SCC cell lines and tumor tissue, and is predictive of poor outcome [12]. The abundance of PKCι mRNA predicted sensitivity to an anti-rheumatoid agent, aurothiomalate, in a panel of lung cancer cell lines [8]. Auranofin, a gold complex used to treat rheumatoid arthritis was shown to inhibit the PI3K/AKT/mTOR signaling in NSCLC cell lines. The administration of auranofin to mice with xenograft tumors significantly suppresses tumor growth without inducing toxic effects [13]. It is of interest that auranofin enhances ibrutinib activity in EGFR mutant LUAD by inhibiting the expression or phosphorylation of multiple key nodes in AKT/mTOR and MEK/ERK pathways [14]. Furthermore, it has been demonstrated that PKCι plays an important function in KRAS LUADs [15, 16]. PAK1 is a Ste20 (MAP 4 K) member that is frequently overexpressed or amplified, and has a critical function in cell growth migration, invasion and apoptosis in NSCLC [17]. PAK1 confers cisplatin resistance in NSCLC patients [18]. PAK1 signaling has been shown to cause resistance to MAPK kinase inhibitors in BRAF mutant melanomas [19]. PAK1 mRNA expressing EGFR mutant tumors are resistant to EGFR tyrosine kinase inhibitors. The combination of gefitinib with an AKT inhibitor (perifosine) or PAK1 inhibitor (IPA-3) almost completely suppresses the tumor burden in nude mice harboring gefitinib resistant cells [20]. Together with others, we have demonstrated that the combination of AKT and EGFR inhibitors could be of benefit in patients with EGFR mutations [21] [22], however, even when blocking AKT, tumor regrowth could occur through the activation of other downstream regulators, via Src/FAK [22, 23]. We posit that different classes of LUADs, such as EGFR mutant LUADs, KRAS mutant LUADs and SCCs, could be sensitive to the inhibition of PKCι with auranofin plus a PAK inhibitor. Several attempts have been made to specifically target PAK1 in cancer, however, its catalytic pocket is large and highly flexible, in addition to its highly mobile N-terminal lobe, which presented a challenge in preventing specific PAK inhibitors [24, 25]. For this reason, we used IPA-3 (2,2′-dihydroxy-1,1′-dinaphthyldisulfide) as a PAK inhibitor [26] with PKCι inhibitor.

This study aimed to estimate the therapeutic effect of PKCι-PAK1 signaling pathways in different types of NSCLC.

## Materials and methods

Detail of standard methodologies for cell culture, reagents, cell viability assay, colony formation assay, and western blotting analysis are described in Supplementary materials and methods.

### In vitro screening for PAK1 inhibitor

The measurement of PAK1 kinase was performed on a Caliper LabChip EZ Reader II equipped with a 12-sipper chip in Profiler Pro separation buffer supplemented with CR-8 and analyzed using EZ Reader software (Caliper Life Sciences; Hopkinton, MA, USA). The test compounds were incubated with PAK1 kinase and fluorescence-labeled substrate Peptide 14 (Sequence RRRLSFAEPG) in the kinase assay buffer at 30 °C for 10 min, followed by adding 9.5 μM ATP to initiate the reaction. After incubation for 1 h at 30 °C, the phosphorylated and unphosphorylated substrates were separated and detected by EZ Reader II device. IPA-3 was used as a positive control. The separation of peptide 14 was performed under the following optimized conditions: upstream voltage = − 500 V, downstream voltage = − 2400 V and pressure = − 1.4 psi using a marker dye consisting of unphosphorylated peptide 14. After PAK1 inhibitors were screened from the Target Mol-Inhibitory-Library, the anti-tumor effects of candidates were estimated by MTT assay using A549 cell lines.

### Nude mouse xenograft

Four to five-week-old female nude mice were kept in individually ventilated cages (5 per cage) with access to food and water, at 20 °C and 50 ± 20% relative humidity under a 12:12 h light-dark cycles and pathogen free conditions. Three cell line xenograft tumors were established by subcutaneously injecting 4 × 10^6^ cells suspended in phosphate-buffered saline mixed 1:1 with Corning Matrigel (356,237, Corning, NY, USA) via right flank. Tumor size was measured in two orthogonal directions using calipers every 2 days and weights were determined every 2 days. After tumors became palpable (~ 100–300 mm^3^), mice were randomized into a vehicle group and treated groups with IPA-3 alone, auranofin alone, OTSSP167 alone, IPA-3 plus auranofin, or OTSSP167 plus auranofin. Each reagent was suspended in 1% [weight/volume (w/v)] Kolliphor HS15 and administered once daily by intraperitoneal administration (IPA-3 and auranofin) or oral gavage (OTSSP167) of 10 mg/kg. Mice in the untreated group were given the same volumes of 1% Kolliphor HS15. The tumor volume (mm^3^) was estimated using the equation length × (width)^2^ × 0.5.

### Statistical analysis

In MTT and colony forming assays, the strength of interaction between reagents was determined by calculating the combination index (CI) according to method of isobologram-combination index (Chou-Talalay method [27]). The CI < 0.6, 0.6 < CI < 0.8, 0.8 < CI < 0.9, 0.9 < CI < 1.1, 1.1 and > 1.1 indicates strong synergism, moderate synergism, slight synergism, additive interaction, and antagonism, respectively. In mice xenograft experience, volume and weight of tumor and mice body weight were compared as continuous variables by using Mann-Whitney U tests. Two-sided statistics was employed and a *p* value of less than 0.05 was regarded as significant.

## Results

### The combination of a PAK1 inhibitor plus antitumoral compounds showed cell viability and colony formation inhibition synergy

**Fig. 1:**
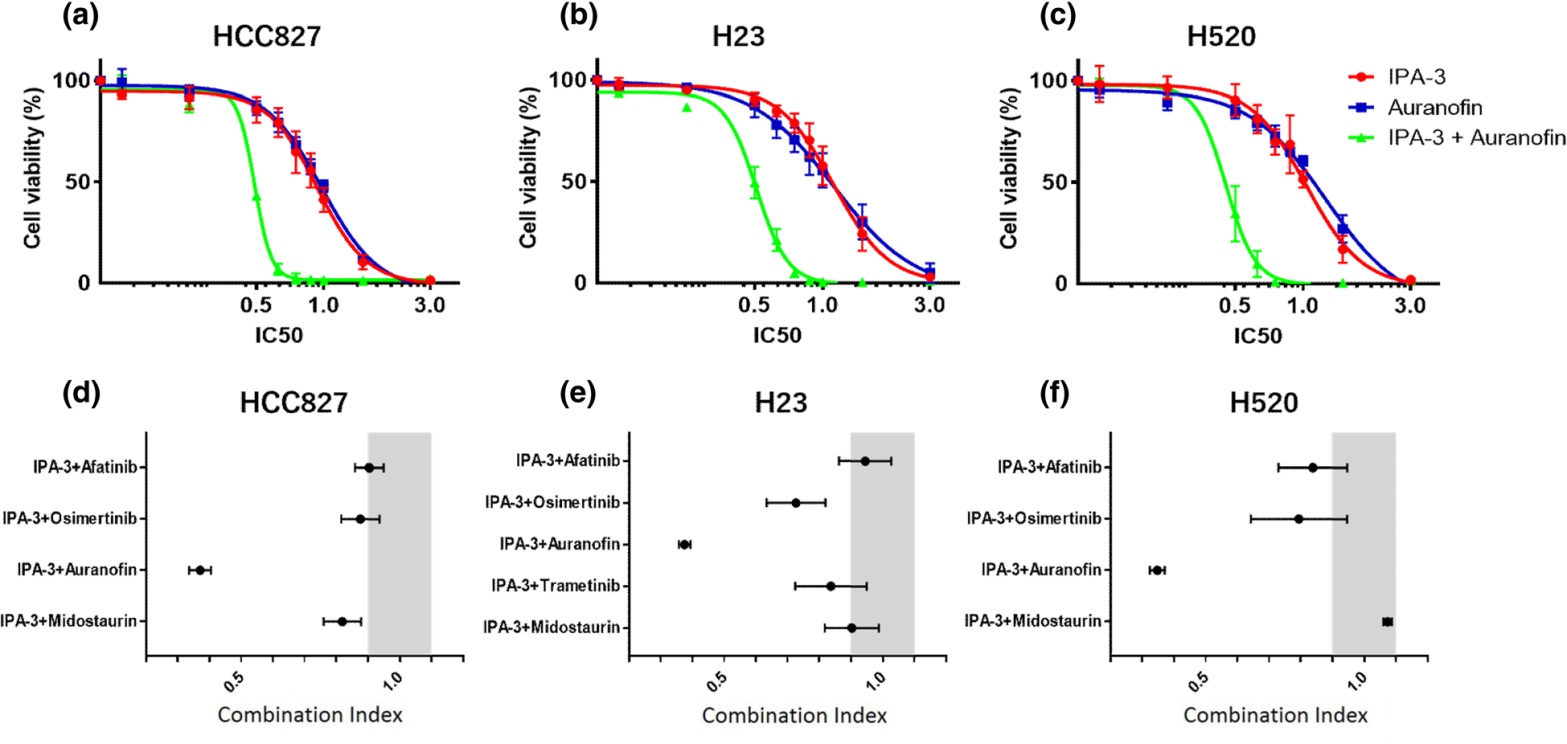
Cell viability and combination index by MTT assay testing single or combination treatment. **a**-**c** The effect in cell viability of the combination of the PAK inhibitor IPA-3 and the PKCι inhibitor auranofin was assessed by MTT assay in HCC827, H23 and H520 cell lines. The experiments were performed by triplicate. Drug concentration was increased gradually from 0 to 3 times of IC50. Cell viability on combination treatment (green line) was decreased significantly in comparison to single drug treatment (red and blue line). **d**-**e** Range of combination index of combination treatment on each cell line. Gray part is the area corresponding to addictive interaction. The combination of IPA-3 plus auranofin was synergic and showed lowest combination index (0.34–0.39) in 3 cell lines. IPA-3 plus midostaurin was synergic only in HCC827 and IPA-3 plus osimertinib was synergic only in H23

To investigate the potential antitumoral properties and possible synergistic effect of a PAK inhibitor (IPA-3) combination with several compounds used in cancer treatment, such as EGFR inhibitors (osimertinib and afatinib), PKCι inhibitor (auranofin), MEK inhibitor (trametinib), and a Flk3/Syk, and potential multiple PKC inhibitor (midostaurin), several lung cancer cell lines possessing different molecular characteristics were tested, such as HCC827 (*EGFR* mutated), H23 (*KRAS* mutated) and H520 (PAK1 overexpression). We evaluated cell viability and colony formation inhibition. Results indicated that the IPA-3 plus osimertinib combination showed synergism in H23 cell lines in cell viability assays with a CI of 0.73. IPA-3 plus trametinib was also synergistic in the H23 cell line (CI was 0.74) (Fig. 1a). To highlight, IPA-3 plus PKCι inhibitor, auranofin, showed the highest synergism in each of the 3 cell lines (CI ranged 0.34 to 0.39) regardless of difference in histology or genetic profile (Fig. 1). IPA-3 plus midostaurin was slightly synergistic or addictive in 3 cell lines. The CIs of each combination treatment were shown in Table 1. The IC50s of each inhibitor in 3 cell lines are shown in Additional file 1: Table S1.

**Table 1:** Combination index of regents in 3 cell lines

	IPA-3 + Afa	IPA-3 + Osi	IPA-3 + Aura	IPA-3 + Mido	IPA-3 + Tra	Osi + Aura
HCC827	0.951	0.876	0.371	0.819		1.123
H23	0.944	0.727	0.376	0.902	0.836	0.929
H520	0.838	0.794	0.347	1.073		0.766

Abbreviations: *Afa* afatinib, *Aura* auranofin, *Mido* midostaurin, *Osi* osimertinib, *Tra* trametinib

**Fig. 2:**
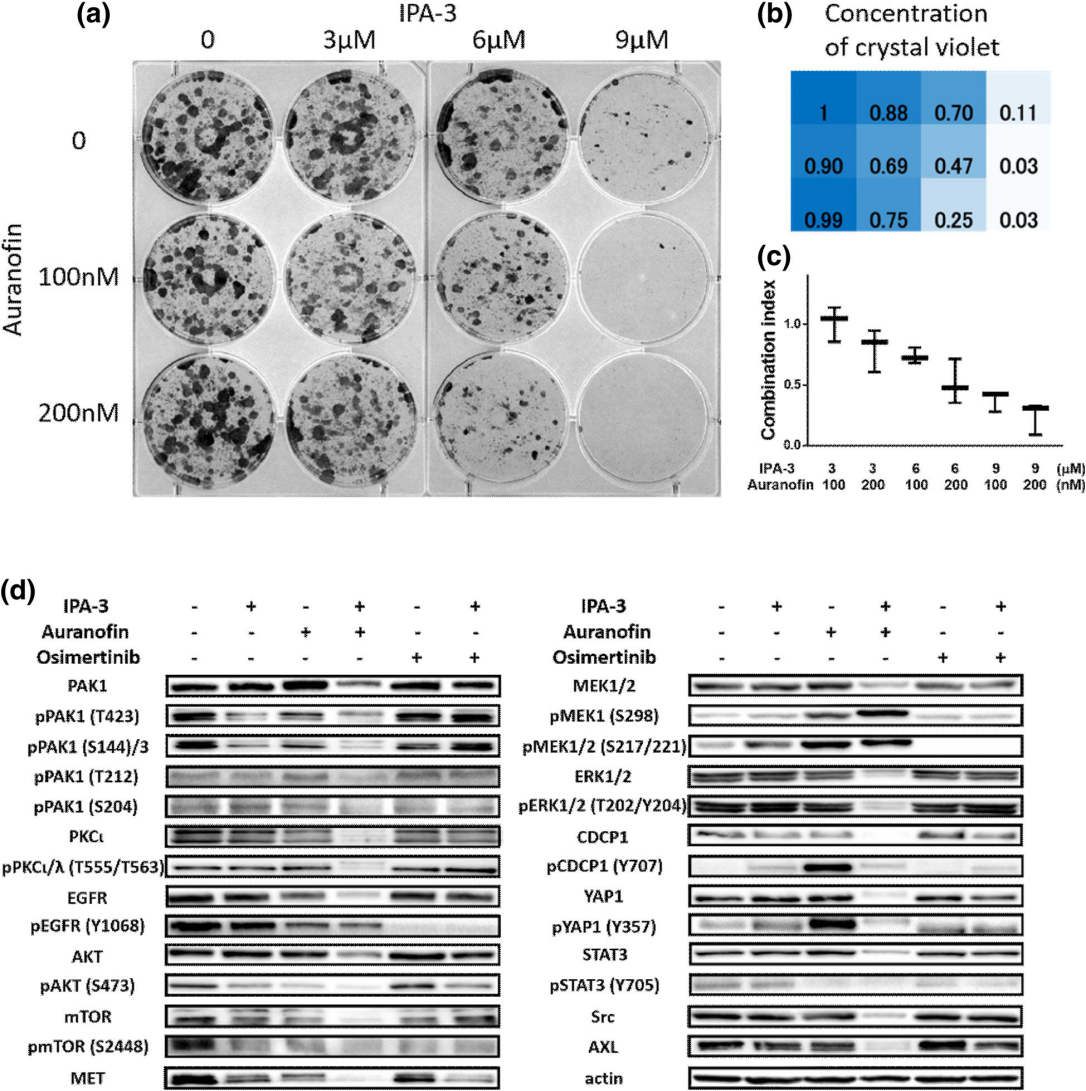
Cell viability by colony forming assay and western blotting by treatment using single reagent or combination of IPA-3 plus auranofin in HCC827 cell lines. IPA-3 plus auranofin potentiates colony formation inhibition and protein expression. **a** HCC827 cell colonies were grown under single or combination increasing dose of IPA-3 and/or auranofin. Fixed colonies were stained using crystal violet. The experiments were made at least three times and a representative image of colon formation assay is shown. **b** Concentration of crystal violet was represented as ratio to control (non-treatment colonies defined as 1). Crystal violet was absorbed using 2% sodium dodecyl sulfate and measured at 570 nm. **c** Combination index by each concentration of IPA-3 and auranofin. Combination index decreased in dose-dependent manner. Combination index was included in synergistic area (< 0.9). **d** HCC827 cell line was exposed to 50 μM IPA-3, 15 μM auranofin or 0.1 μM osimertinib and the combinations of IPA-3 plus auranofin or osimertinib for 6 h. Protein expression and activation were analyzed by western blotting. Actin was used as house-keeping protein. The experiments were made at least twice. Abbreviations: S, serine; T, threonine; Y, tyrosine

Furthermore, dose-dependent colony formation assays indicated that the combination of IPA-3 plus auranofin significantly inhibited the forming of colonies, compared to single agent treatment and showed high synergism in 3 cell lines (lowest CI ranging from 0.24–0.46) (Fig. 2 and Additional file 1: Figure S1). The values of quantification of crystal violet concentration are shown in Fig. 2b and Additional file 1: Figure S1B/S1E. The combination index value was calculated to assess the nature of drug-drug interactions and it decreased in a dose-dependent manner (Fig. 2c and Additional file 1: Figure S1C/S1F).

### The combination of a PAK1 inhibitor plus antitumoral compounds downregulated the expression of proteins related to deregulated signaling pathways in lung cancer

To study the mechanisms involved in the synergism between IPA-3 and auranofin, we analyzed the effects of this combination in the expression and phosphorylation of proteins that belong to several signaling pathways related to lung cancer. Cell lysates were harvested after being treated with the indicated drug concentrations for 6 h and subjected to western blotting analysis. Results showed that the combination of IPA-3 plus auranofin abrogated the expression of total/phosphorylation of PAK1, PKCι, ERK 1/2, mTOR, AKT, and YAP1 in the 3 lung cancer cell lines studied HCC827 (EGFR-mutant LUAD), H23 (KRAS-mutant LUAD) and H520 (PAK1 overexpression). Also, downregulation of protein expression of EGFR, MEK, AXL, Src and STAT3 was observed. (Fig. 2 and Additional file 1: Figure S2). In summary, there is a clear downregulation of proteins, and, in most of them, a clear decrease in phosphorylation was detected when combining IPA-3 plus auranofin, indicating that this drug combination may target multiple signaling pathways. In addition, we investigated the molecular effects of auranofin combined with osimertinib, an EGFR inhibitor used in EGFR-mutated lung cancer patients. The drug combination was tested in the EGFR-mutated cell line, HCC827, at the indicated dose for a period of 6 h. Protein lysates were analyzed by western blotting technique which showed a clear protein expression decrease in AKT, MET, AXL and CDCP1 (Fig. 2). We carried out similar experiments with the combination of auranofin plus trametinib, a MEK inhibitor, in the KRAS mutated cell line, H23, and observed a clear downregulation of PAK1, PKCι, EGFR, ERK 1/2, AKT, MET, AXL, STAT3, and MEK 1/2. Inhibition of phosphorylated PAK1 (Ser144), PKCι, ERK 1/2, STAT3 and MEK 1/2 was also observed.

### Screening of alternative PAK1 inhibitor and validation of combination therapeutic effect with auranofin

**Fig. 3:**
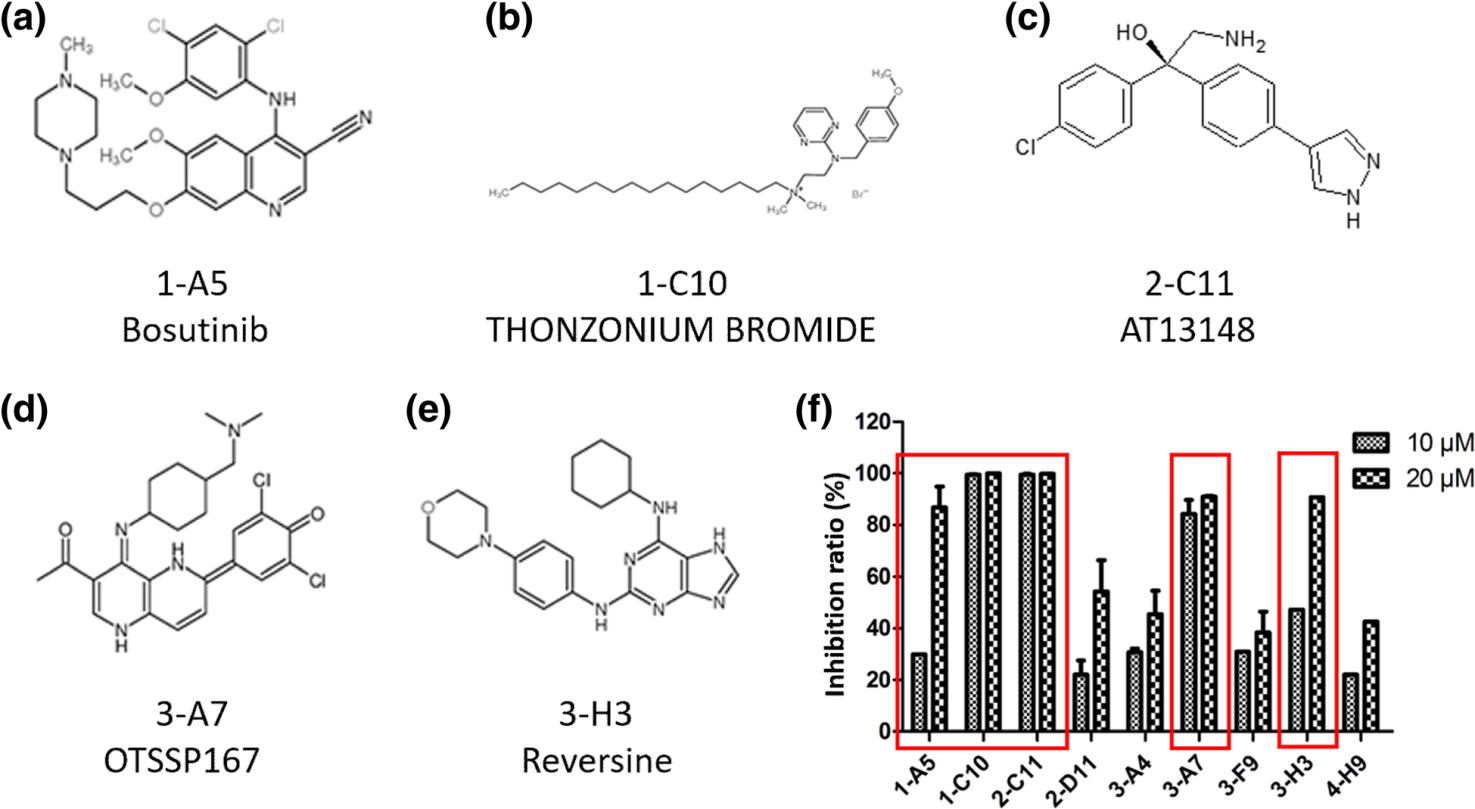
Structures and cell growth inhibitions of the selected PAK1 inhibitors tested in A549 cell lines based on the preliminary screening (Additional file 1: Figure S4). The effect in cell viability of the potential PAK inhibitors. A549 cells were incubated with the 9 compounds at the concentration of 10 or 20 μM for 72 h and the cell viability was determined using MTT assay. Five compounds (1-A5, 1-C10, 2-C11, 3-A7, and 3-H3) significantly suppressed the growth of A549 cell lines at the range concentration of 10 and 20 μM. Statistical analysis was performed by comparing with the value of control. The experiments were made by triplicate. **a**-**e** The structures of 5 compounds which suppressed the cell viability. **f** Cell inhibition ratio on tested 9 compounds. Five compounds surrounded red square showed high PAK1 inhibition

To identify pharmacological inhibitors of PAK1 kinase activity, we screened the Target-Mol inhibitor library based on an optimized LabChip EZ Reader kinase assay system. Titration and validation of the kinase assay are shown in Additional file 1: Figure S3. Out of 320 compounds tested, 13 candidates showed PAK1 inhibitory effect. The details and inhibition ratio of each compound are shown in Additional file 1: Table S2 and Additional file 1: Figure S4. Among them, the PAK1 inhibitory effects of some compounds, such as bosutinib (1-A5), WH-4-023 (3-F9), and MHY1485 (4-H9), were comparable to IPA-3, and their inhibitory effect on cell growth was estimated using the MTT assay. Cell viability tested in A549 cell lines showed that 5 compounds (bosutinib, thonzonium bromide, AT13148, OTSSP167, and reversine) significantly suppressed cell growth at the concentrations of 10 and 20 μM (Fig. 3).

**Fig. 4:**
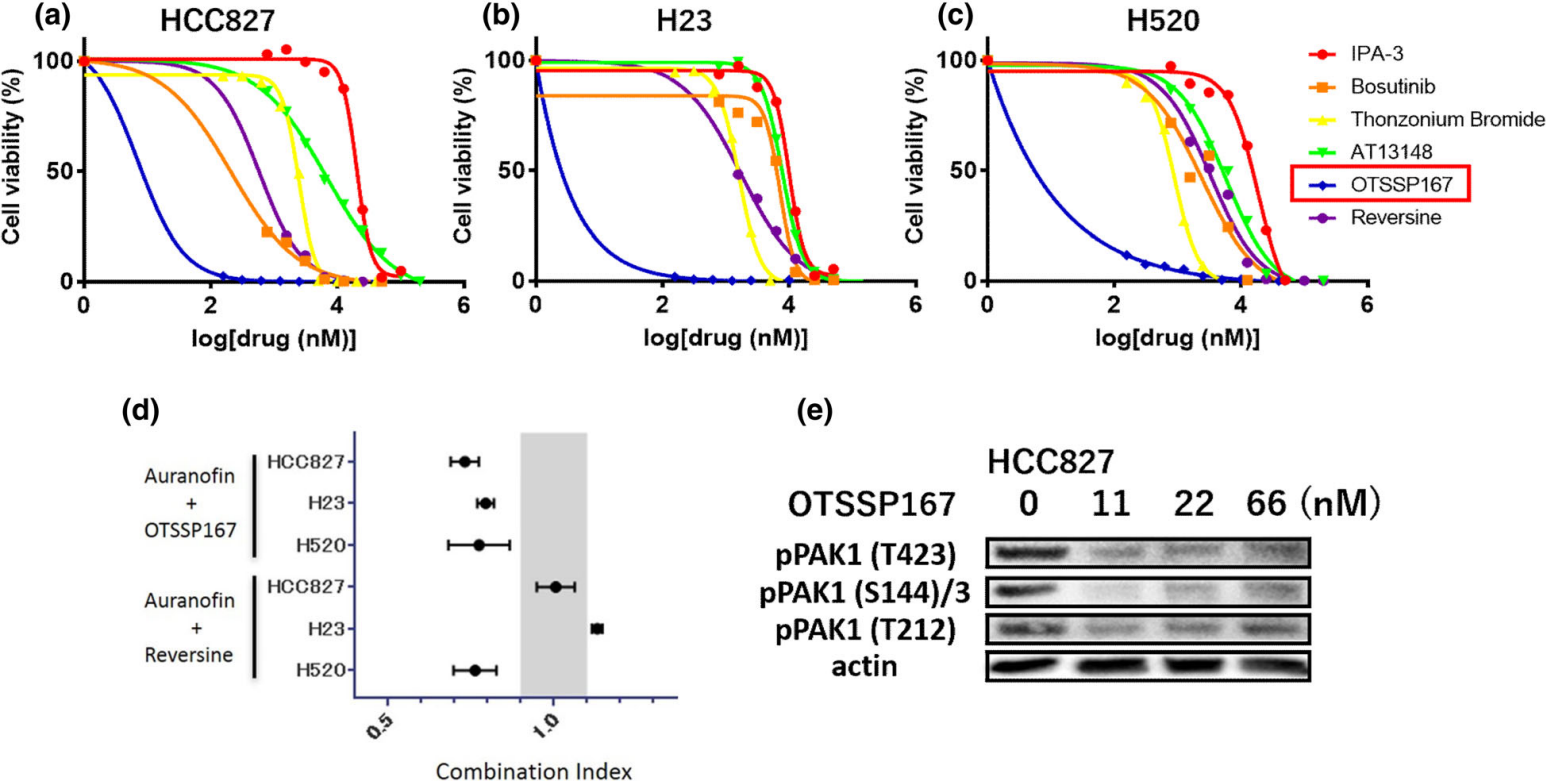
Cell viability assay using potential PAK inhibitors. The potential PAK inhibitor OTSSP167 synergizes with auranofin in cell viability assays. **a**-**c** The results of MTT assay using alternative PAK1 inhibitors candidates in 3 cell lines: HCC827 (**a**), H23 (**b**), and H520 (**c**). OTSSP167 showed the lowest IC50 in 3 cell lines (blue line). (**d**) Combination index of OTSSP167 plus auranofin indicated synergism in 3 cell lines. The combination of reversine plus auranofin also indicated synergism in H520 cell lines but not in HCC827 and H23 cell lines. (**e**) OTSSP167 inhibited 3 phosphorylated PAK1 residues at the concentration of 0.5, 1.0, and 3.0 times higher of IC50

**Fig. 5:**
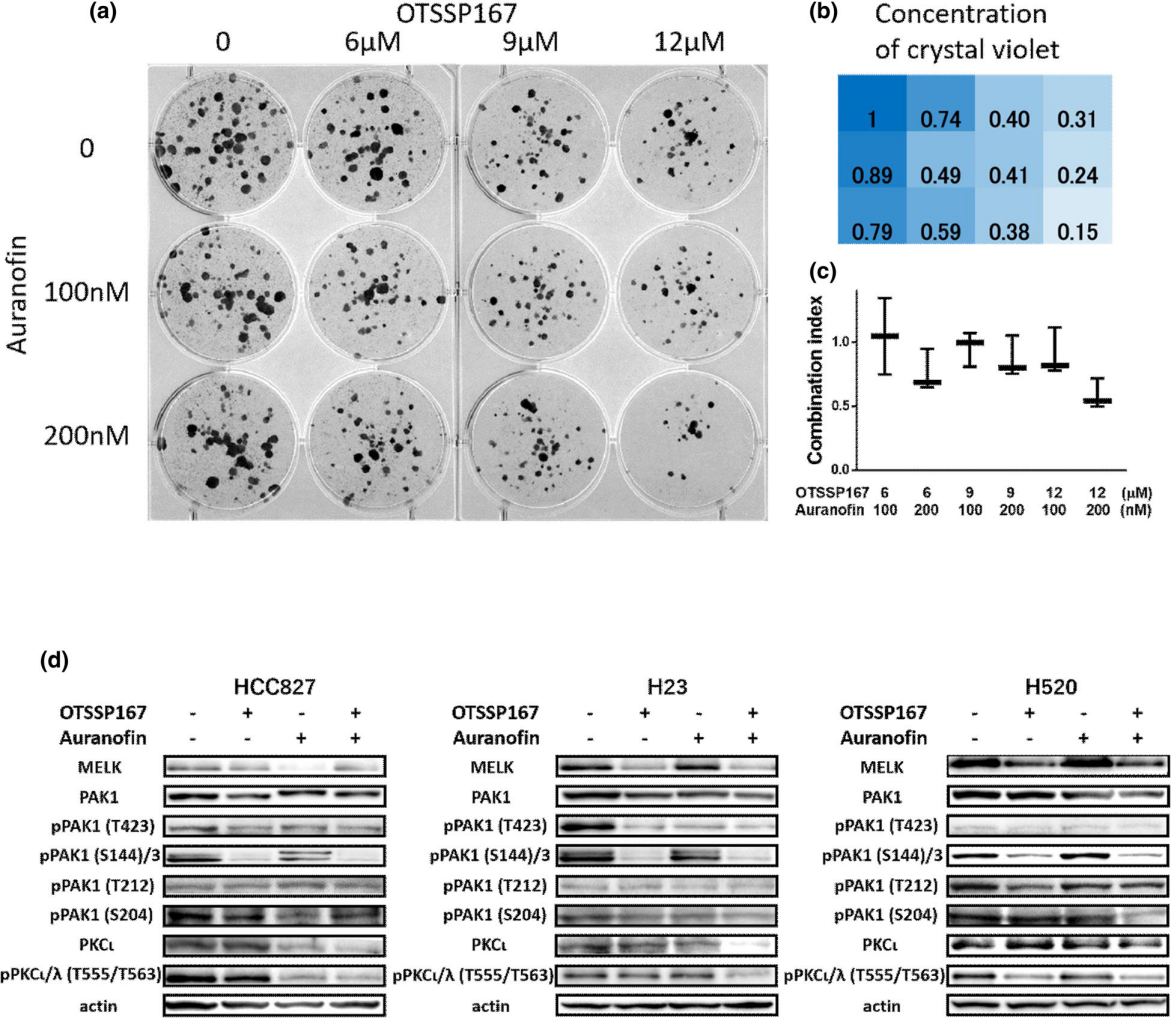
Colony formation assay in HCC827 cell lines and western blotting in 3 cell lines by mono therapy or combination treatment of OTSSP167 plus auranofin. The combination of OTSSP167 and auranofin potentiates colony formation inhibition in HCC827 cell lines. Combination of OTSSP167 and auranofin downregulates expression and inhibits activation of PAK1 and PKCι. **a** HCC827 cell colonies were grown under single or combination treatment (OTSSP167 and auranofin) at indicated dose. Fixed colonies were stained using crystal violet. **b** Concentration of crystal violet was represented as ratio to control (non-treatment colonies defined as 1). Crystal violet was absorbed using 2% sodium dodecyl sulfate and measured at 570 nm. **c** Combination index by each concentration of OTSSP167 and auranofin. Combination index decreased in dose-dependent manner. Combination index was included in synergistic area (< 0.9). **d** HCC827, H23 and H520 cell lines were exposed to 3 or 10 μM auranofin (10 μM for HCC827, 3 μM for H23 and H520) or 1 μM OTSSP167 and the combination of OTSSP167 plus auranofin for 6 h. Protein expression and activation were analyzed by western blotting. Actin was used as house-keeping protein. The experiments were made at least twice. The combination of OTSSP167 plus auranofin abrogated the expression and phosphorylation of PAK1 and PKCι. Abbreviations: S, serine; T, threonine; Y, tyrosine

Furthermore, among 5 potential PAK1 inhibitors, OTSSP167 plus auranofin showed synergism in 3 cell lines. CIs in MTT assay were 0.78–0.79. The protein expressions of phosphorylated PAK1 were suppressed by × 0.5, × 1.0, and × 3.0 times higher dose of IC50 (Fig. 4). Reversine indicated synergism with auranofin only in H520 cell lines (CI: 0.76). The IC50 and CI of OTSSP167 or reversine plus auranofin are shown in Table 2. Colony formation assay showed synergistic effect of OTSSP167 plus auranofin in 3 cell lines with lowest CI ranging from 0.50–0.59 (Fig. 5a-c and Additional file 1: Figure S5). Western blotting experiments showed that OTSSP167 inhibited more PAK1 phosphorylated residues compared to IPA-3. The combination of OTSSP167 plus auranofin abrogated phosphorylated PAK1 (Thr432 and Ser144 in 3 cell lines) (Fig. 5d). In addition, the combination of these two drugs downregulated the PKCι expression and inhibited its phosphorylation.

**Table 2:** IC50 (nM) or combination index (CI) of each single treatment or combination treatment in 3 cell lines

	Reagent	HCC827	H23	H520
IC50	OTSSP167	22	18	13
	Reversine	350	1300	5300
CI	OTSSP167 + Aura	0.777	0.795	0.783
	Reversine + Aura	1.063	1.130	0.795

Abbreviations: *Aura* auranofin

### The combination of PAK1 and PKCι inhibitors suppressed tumorigenesis in a nude mouse Xenograft model

**Fig. 6:**
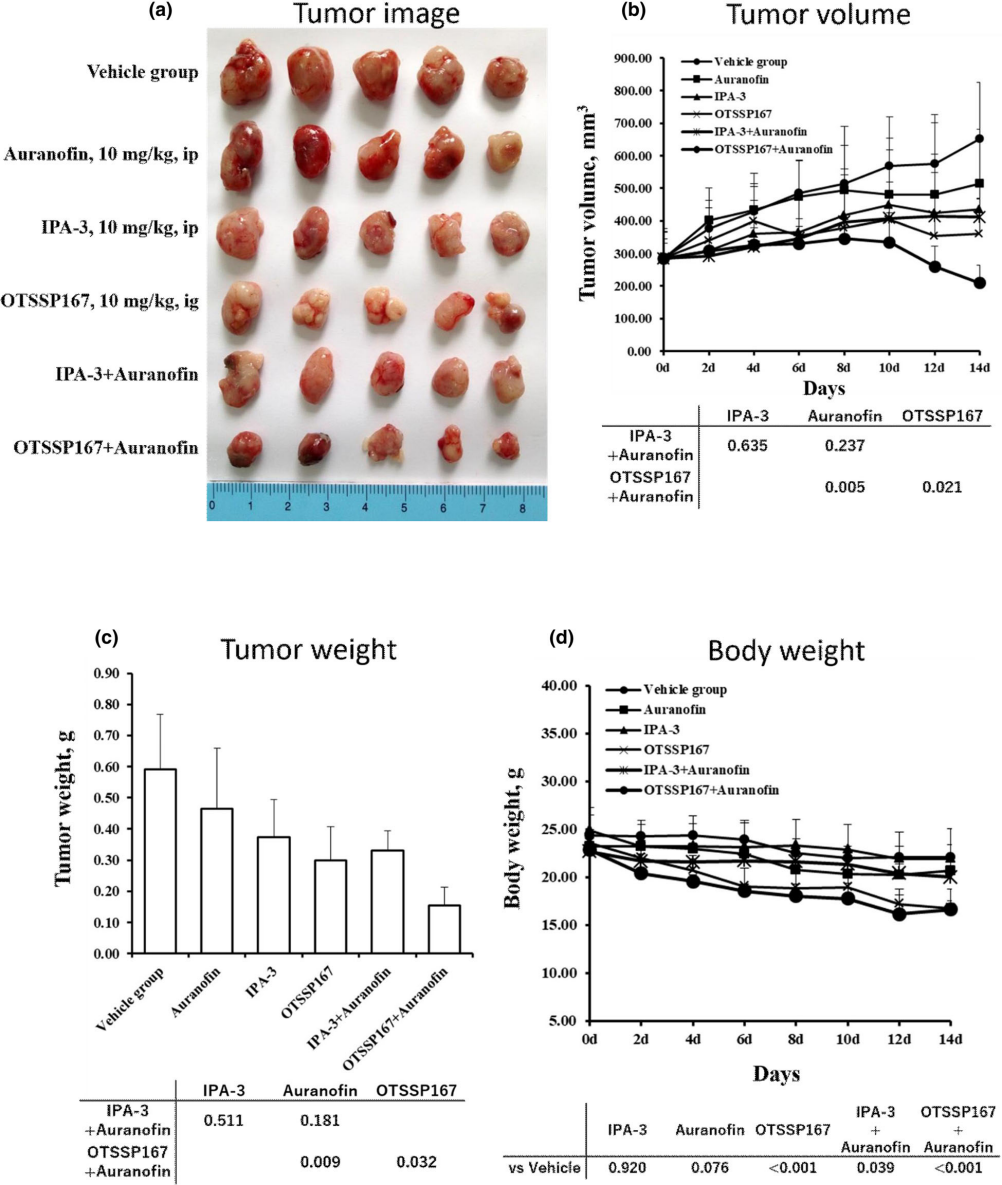
Anti-tumor effects of single reagent or combination treatment using IPA-3, auranofin, and OTSSP167 in nude xenograft model in HCC827. **a** HCC827 cell lines were used for the xenograft model. Mice were treated once daily with the PAK1 inhibitor (IPA-3 or OTSSP167) single agent or combined with auranofin at given concentrations. Representative images of tumors measured at the end of the study compared with the control group are shown. **b** Line graphs of tumor volumes after treatment by each therapeutic course (vehicle, single reagent, or combination). Tumor volumes were recorded every 2 days until 14th or 26th day. Errors bars mean + − SD of 5 animals per group. *P* values by comparing tumor volumes are described in the table. **c** Histographs of tumor weight treated by each therapeutic course (vehicle, single reagent, or combination) at the time of sacrifice. *P* values by comparing tumor weight are described in the table. **d** Line graphs of mice weight on every 2 days of each treatment course (vehicle, single reagent, or combination). *P* values by comparing mice body weight are described in the table.

We performed nude mice xenograft experiments to explore the pharmacological combined effect of PAK1 and PKCι inhibitors. HCC827, H23, or H520 cell lines were inoculated subcutaneously into mice and when tumor size reached an average volume of 100–300 mm^3^, single reagent (IPA-3, OTSSP167, auranofin) or combination of IPA-3 or OTSSP167 plus auranofin or vehicle were inoculated via intraperitoneal administration (auranofin and IPA-3) or oral gavage (OTSSP167) every 2 days for 2 or 4 weeks. Combination therapy showed stronger anti-tumor effect compared to mono therapy in 3 mice model cell lines. IPA-3 plus auranofin inhibited tumor growth, both in volume and weight, compared to IPA-3 alone or auranofin alone. The combination of OTSSP167 plus auranofin showed significant inhibition of tumor growth in the 3 mice model cell lines compared to single OTSSP167 or auranofin treatment (Fig. 6 and Additional file 1: Figure S6). Compared to vehicle group, mice body weight was significantly reduced in OTSSP167 plus auranofin and single OTSSP167 treatment in the 3 mice model cell lines. The combination of IPA-3 plus auranofin induced significant body weight loss in HCC827 and H520 cell lines mice models.

## Discussion

Inactive PAK1 is reported to be a homodimeric protein. Binding of Cdc42/Rac1 to Cdc42/Rac1-binding domain (CRIB) relieves inhibition by disrupting the PAK1 homodimer [28]. PAK1 is activated by several mechanisms that include PKCι commonly upregulated in NSCLC [12, 29], such as TNFα, CD3/CD28 (T cell receptor engagement). EGFR signaling actively suppresses TNF mRNA levels by inducing expression of miR-21. EGFR TKIs result in loss of miR-21 and increase TNF mRNA stability, leading to EGFR TKI resistance in EGFR mutant NSCLC [30]. Therefore, it is tempting to speculate that an EGFR TKI, PAK inhibitor combination could serve as novel combinatory therapy for EGFR mutant NSCLC. The development of PAK1 inhibitors is difficult due to their large and highly flexible catalytic pocket and highly mobile N-terminal lobe [29, 31, 32]. PAK inhibitor combinations with targeted drugs have been tested in NSCLC cell lines, including apoptosis protein inhibitors (IAP, EGFR, MEK1/2 and Src inhibitors with PAK1 knockdown) [33]. Strong combinatorial activity was confirmed for dual inhibition of PAK1 and IAP in EBC-1 cells [33]. Activity was demonstrated with an inhibitor targeting PAK1 activation-3′ (IPA-3) [33]. However, the cardiac toxicity noted with IPA-3 prevents its clinical use [34]. PAK1 serves as a mediator of intracellular calcium ion homeostasis in the heart. PAK1 inhibition induces exaggeration of calcium ion and arrhythmia [35, 36]. Although it is unknown whether the toxicity is reversible or not, the risk can be reduced by lower doses of IPA-3 [34]. An auranofin phase I study for rheumatoid arthritis patients [37] and a randomized multicenter study for asthma patients [38] have reported no cardiac toxicity. Our study suggested that the dose of IPA-3 can be decreased in combination with auranofin. Nevertheless, cardiac toxicity is a major concern and attention should be paid to safety use in PKCι-PAK1 signaling strategy. We tested the combination of IPA-3 plus other inhibitors in cell models. IPA-3 plus auranofin showed high synergism in cell viability and colony formation assays in three NSCLC cell lines used. The inhibitory effect was superior to other combinations, such as EGFR tyrosine kinase inhibitors (either osimertinib or afatinib) in the HCC827, or MEK inhibitor (trametinib) in the H23 cell line. Also, in the SCC cell line, the combination index was significant for IPA-3 plus auranofin. Auranofin has already been clinically available for rheumatoid arthritis and a clinical trial for NSCLC and small cell lung cancer is ongoing (NCT01737502). Another PKCι inhibitor, gold compound aurothiomalate, is also clinically available for rheumatoid arthritis and a phase I study has been successfully performed for advanced NSCLC [39]. However, IPA-3 has never been clinically used so we explored a PAK1 inhibitor with potential clinical availability.

OTSSP167 is a maternal embryonic leucine-zipper kinase (MELK) inhibitor with anti-cancer effect reported in several tumors, as well as in chronic lymphocytic leukemia [40]. The anti-tumor effects of OTSSP167 have been investigated in breast cancer (NCT02926690) and leukemia (NCT02795520) clinical trials [40–42]. OTSSP167 [41] inhibited PAK1 with lower IC50 than other potential PAK1 inhibitors, such as bosutinib [43], thonzonium bromide [44], AT13148 [45] and reversine [46] (Fig. 4) and showed high synergism with auranofin in all three cell lines.

The phosphorylated residue Ser144 of PAK1 was inhibited in three cell lines. The phosphosites, Ser21 or Thr423, also contribute towards PAK1 activation, although, Ser144 is the most critical for PAK kinase activity [47]. Phosphorylated Ser144 on PAK1 was abrogated by IPA-3 plus auranofin, as well as by OTSSP167 plus auranofin.

Our model showed that some PAK1 phosphosites were inhibited by, not only IPA-3, but also auranofin alone. IPA-3 did not inhibit PKCι expression. This suggests that auranofin inhibits PAK1 from the upper stream of PKCι-PAK1 signaling and stronger inhibition of PKCι-PAK1 signaling resulted in stronger synergism. Although osimertinib monotherapy in HCC827 cell lines or trametinib monotherapy in H23 cell lines also inhibited some phosphorylated PAK1, the combination of auranofin plus these reagents was not synergistic and showed less abrogation of RTK and non-RTK. In addition, midostaurin, a FLT3 inhibitor, and a potential inhibitor of all PKC isoforms [48] was not synergistic with auranofin (Fig. 1). In addition, although OTSSP167 served as a MELK inhibitor, inhibition of MELK expression was similar between OTSSP167 alone, and OTSSP167 plus auranofin (Fig. 5d), suggesting that synergism in OTSSP167 plus auranofin was not induced by MELK inhibition. Thus, PKCι-PAK1 signaling is an important pathway in tumor genesis in EGFR mutant, KRAS mutant and SCC cell lines. Additionally, due to the inhibition of most proteins analyzed by the auranofin plus IPA-3 or OTSSP167 combination, we hypothesize that this compound combination could affect the proteasome activity as previously reported [49].

In the mice xenograft model, a significant decrease in tumor volume was confirmed by the OTSSP167 plus auranofin combination. Although IPA-3 plus auranofin showed anti-tumor effect, it did not reach a significant difference. OTSSP167 suggested stronger PAK1 inhibition compared to IPA-3 in western blotting; phosphorylated PAK1 at Thr423/Ser144/Thr204 in HCC827 cell lines and Thr423 in H23 cell lines were inhibited by OTSSP167 alone, but not by IPA-3 alone. These differences between IPA-3 and OTSSP167 might explain the difference in results of the mice xenograft model. Although mice body weight was significantly decreased in OTSSP167 alone, or OTSSP167 plus auranofin in all 3 cell lines, there was no significant difference between OTSSP167 alone, and OTSSP167 plus auranofin. Chung et al. showed OTSSP167 was tolerable in the mice model without adverse body weight loss [41]. Adverse events of OTSSP167 should be further examined.

Our research has centered on targeting PKCι-PAK1 signaling and was effective with auranofin plus OTSSP167 in 3 lung cancer models in vitro and in vivo*.* Although OTSSP167 inhibited phosphorylated PAK1, we cannot rule out that other activators of PAK1 can intervene in lung cancer, like MLK3 and TNFα.

Auranofin has been investigated in a clinical trial for NSCLC and small cell lung cancer (NCT01737502). A Phase I study of OTSSP167 for solid tumors (NCT01910545) and a feasibility evaluation for healthy volunteers (NCT02768519) have been completed. Other clinical trials of OTSSP167 are ongoing in breast cancer (NCT02926690) and leukemia (NCT02795520). Targeting PKCι-PAK1 signaling pathways is of interest to be further tested clinically in *EGFR* mutant, *KRAS* mutant, and squamous NSCLC patients.

## Conclusions

The Combination of PKCι-PAK1 inhibitors was highly synergistic in EGFR and KRAS mutant adenocarcinoma and squamous cell carcinoma of the lung, in both in vitro and in vivo mice models. It is warranted to further test the therapeutic strategy of targeting PKCι-PAK1 signaling pathways in EGFR mutant, KRAS mutant and squamous NSCLC patients.

## Additional file

**Additional file 1: Table S1.** Profiles of cell lines and IC50 (nM) of each reagent. **Table S2.** The profiles of tested potential PAK1 inhibitors. **Figure S1**. IPA-3 and auranofin potentiates colony formation inhibition in H23 and H520 cell lines. **Figure S2**. Western blotting by treatment using single reagent or combination of IPA-3 plus auranofin in H23 and H520 cell lines. **Figure S3.** Optimization and validation of the kinase assay for PAK1 inhibitor screening. **Figure S4.** Summary of cell growth inhibition using potential PAK1 inhibitors. **Figure S5.** OTSSP167 plus auranofin potentiates colony formation inhibition in H23 and H520 cell lines. **Figure S6.** Anti-tumor effects of single reagent or combination treatment using IPA-3, auranofin, and OTSSP167 in nude xenograft model in H23 and H520 cell lines.

## Data Availability

Data is shown properly in main manuscript and supplemental files. No additional data is provided through the special link.
