# Supplementary material for: Targeting PKCι-PAK1 signaling pathways in EGFR and KRAS mutant adenocarcinoma and lung squamous cell carcinoma
Source: Cell Commun Signal. 2019 Oct 28;17:137. doi: 10.1186/s12964-019-0446-z (PMC6819333; doi:10.1186/s12964-019-0446-z)
Supplement: Supplementary file 1 — Additional file 1: Table S1. Profiles of cell lines and IC50 (nM) of each reagent. Table S2. The profiles of tested potential PAK1 inhibitors. Figure S1. IPA-3 and auranofin potentiates colony formation inhibition in H23 and H520 cell lines. Figure S2. Western blotting by treatment using single reagent or combination of IPA-3 plus auranofin in H23 and H520 cell lines. Figure S3. Optimization and validation of the kinase assay for PAK1 inhibitor screening. Figure S4. Summary of cell growth inhibition using potential PAK1 inhibitors. Figure S5. OTSSP167 plus auranofin potentiates colony formation inhibition in H23 and H520 cell lines. Figure S6. Anti-tumor effects of single reagent or combination treatment using IPA-3, auranofin, and OTSSP167 in nude xenograft model in H23 and H520 cell lines. [file 12964_2019_446_MOESM1_ESM.pdf]

Supplementary materials and methods. Materials and Methods for Cell culture, Reagents, Cell viability assay, Colony formation assay, and Western blotting analysis

Cell culture

Genetically or histologically different 3 types of NSCLC cell lines were used for estimation of PAK1 inhibitor mono- and combination treatment; HCC827 (LUAD harboring EGFR mutation, exon 19 deletion), H23 (LUAD harboring KRAS mutation, G12C), and H520 (SCC cell carcinoma without EGFR or KRAS mutation but with high PAK1 amplification) were cultured with RPMI medium supplemented with 10% fetal bovine serum and 50 µg/mL penicillin-streptomycin. Cells were maintained in a humidified atmosphere with 5% CO_2 at 37°C. Another cell line, A549 (LUAD harboring KRAS mutation, G12S), was cultured at same condition and used for screening of potential PAK1 Inhibitors.

Reagents

IPA-3 (S7093, Selleckchem, Houston, TX, USA) and auranofin (A6733, Sigma-Aldrich, St. Louis, MO, USA) were used as PAK1 inhibitor and PKCι inhibitor, respectively. In the combination strategy with IPA-3, afatinib (EGFR-tyrosine kinase inhibitor, S1011, Selleckchem, Houston, TX, USA) or osimertinib (S7297, Selleckchem, Houston, TX, USA), and trametinib (MEK inhibitor, S2673, Selleckchem, Houston, TX, USA) were tested as key drugs for LUAD and SCC, respectively. Midostaurin (S8064, Selleckchem, Houston, TX, USA) was also estimated with IPA-3 as multiple PKC inhibitor. Five candidate reagents for substitution of PAK1 inhibitor (bosutinib, thonzanium bromide, AT13148, OTSSP167, and reversine) were kindly provided from Dr. Peng Cao (Nanjing University of Chinese Medicine, Nanjing, China).

Cell viability assay

Cell viability was assessed by the Thiazolyl Blue Tetrazolium Bromide (MTT) assay and the concentration of drug required for 50% growth inhibition (IC50) was determined. Tumor cells were seeded at the number of 2000 (H23) or 3000 (HCC827 and H520) per well in 96-well plates (83.3924, SARSTEDT, Nümbrecht, Germany) and allowed to attach for 24 hours. The cells were treated with medium including inhibitor(s) for 3 days. After treatment, cells were incubated with medium containing MTT (22.5 µg in each well) for 90 minutes at 37°C. Culture medium with MTT was removed and formazan crystals were reabsorbed in 100 µL of dimethyl sulfoxide. Cell viability was determined by measuring the absorbance at 492 nm by a microplate reader (BioWhittaker, Gaitherburg, MD, USA).

Colony formation assay

Three hundred cells were disseminated to each well in 6-well plate (83.3920, SARSTEDT, Nümbrecht, Germany). The cells were cultured to attach for 24 hours and then the medium was replaced with or without inhibitor(s). Medium including inhibitor(s) were changed every 3 days for 10-14 days. After 10-14 days cell culture, the medium was removed and cells were washed with phosphate-buffered saline (PBS). The colonies were fixed with methanol for 25 minutes at 4°C and stained using 0.5% crystal violet for 15 minutes at room temperature. The colonies were washed and photographed. For semiquantitative measurement, the crystal violet was reabsorbed using 2% sodium dodecyl sulfate (SDS) and the absorbance was measured at 570 nm using TECAM i-control (TECAM, Männedorf, Switzerland).

Western blotting analysis

Cultured cells were washed with PBS and lysed in RIPA buffer (9806, Cell Signaling Technology, Danvers, MA, USA). Following centrifugation at 14000 x rpm for 10 minutes at 4°C, the supernatant was collected as the total cell lysate. Briefly, the lysates containing proteins were electrophoresed on 10% SDS-polyacrylamide gel electrophoresis and transferred to polyvinylidene difluoride membranes (10600021, General Electric, CT, USA). All target proteins were immunoblotted with appropriate primary and secondary antibodies using Odyssey blocking buffer (927, LI-COR Biosciences, Lincoln, NE, USA), Phosphoblocker (AKR-103, Cell biolabs, INC., San Diego, CA, USA), or Can Get Signal/PVDF Blocking Reagent (NKB101/NYPBR, TOYOBO LIFE SCIENCE, Tokyo, Japan). The β-actin was used as an internal control. Chemiluminiscent bands were detected and expression level was measured using Image lab 5.0 (BIO-RAD, Hercules, CA, USA). Used antibodies and concentration are shown as follows.

Antibodies used in Western blotting

**Primary Antibody**	**Dilution**	**Company and Catalog number**
Mouse anti-Beta-actin	1:5000	Sigma Aldrich (#A5441)
Rabbit anti-AKT	1:1000	Cell Signaling (#9272)
Rabbit anti-Phospho-AKT (Ser473)	1:1000	Cell Signaling (#9271)
Rabbit anti-AXL	1:1000	Cell Signaling (#8661)
Rabbit anti-CDCP1	1:1000	Cell Signaling (#4115)
Rabbit anti-Phospho-CDCP1 (Tyr707)	1:1000	Cell Signaling (#13111)
Rabbit anti-EGFR	1:1000	Cell Signaling (#4267)
Rabbit anti-Phospho-EGFR (Tyr1068)	1:1000	Cell Signaling (#3777)
Rabbit anti-ERK1/2	1:1000	Cell Signaling (#9102)
Rabbit anti-Phospho-ERK1/2 (Thr202/Tyr204)	1:1000	Cell Signaling (#9101)
Mouse anti-MEK1/2	1:1000	Cell Signaling (#4694)

Rabbit anti-Phospho-MEK1 (Ser298)	1:1000	Cell Signaling (#9128)
Rabbit anti-Phospho-MEK1/2 (Ser217/221)	1:1000	Cell Signaling (#9121)
Rabbit anti-MELK	1:1000	Cell Signaling (#2274)
Rabbit anti-cMET	1:1000	Cell Signaling (#8198)
Rabbit anti-mTOR	1:1000	Cell Signaling (#2983)
Rabbit anti-Phospho-mTOR (Ser2448)	1:1000	Cell Signaling (#5536)
Rabbit anti-PAK1	1:1000	Cell Signaling (#2602)
Rabbit anti-Phospho-PAK1/2 (Ser144/Ser141)	1:1000	Cell Signaling (#2606)
Rabbit anti-PAK1 (Thr423)/PAK2 (Thr402)	1:1000	Cell Signaling (#2601)
Rabbit anti-PAK1 (Thr212)	1:1000	Abcam (#75599)
Rabbit anti-PAK1 (Ser204)	1:1000	Abcam (#79503)
Rabbit anti-PKCι	1:500	Abcam (#5282)
Rabbit anti-Phospho-PKC iota/lamda (Thr555/Thr563)	1:1000	Abcam (#5813)
Rabbit anti-Src	1:1000	Cell Signaling (#2109)
Rabbit anti-STAT3	1:1000	Cell Signaling (#9363)
Rabbit anti-Phospho-STAT3 (Tyr705)	1:1000	Cell Signaling (#9145)
Mouse anti-YAP1	1:1000	Cell Signaling (#12395)
Rabbit anti-Phospho-YAP1 (Tyr357)	1:1000	Abcam (#62751)

**Secondary Antibody**	**Dilution**	**Company and Catalog number**
Donkey anti-Rabbit Ig HRP-linked	1:2000	GE Healthcare (#NA934)
Sheep anti-Mouse IgG HRP-linked	1:2000	GE Healthcare (#NXA931)

Supplementary Table 1. Profiles of cell lines and IC50 (nM) of each reagent

Cell line	Histology	Mutation	Afa	Osi	IPA-3	Aura	Mido	Tra
HCC827	LUAD	EGFR del 19	0.6	9	22500	3250	300	16000
H23	LUAD	KRAS G12C	800	3200	12500	1200	125	15
H520*	SCC	EGFR-, KRAS-	700	3000	16000	2250	190	15500

*: expressing high level of PAK1

Abbreviations: Afa, afatinib; Aura, auranofin; LUAD, lung adenocarcinoma; Mido, midostaurin; Osi, osimertinib; SCC, squamous cell carcinoma; Tra, trametinib

Supplementary Table 2. The profiles of tested potential PAK1 inhibitors

Index	drug	Formula	Molecular Weight	Bioactivity	Pathways	Receptor	Reference
1-A5	Bosutinib	C26H29Cl2N5O3	530.45	MAPK inhibitor;<br>ABL1, Lyn, HCK, Src inhibitor;<br>BCR inhibitor;<br>Bcr-Abl inhibitor;<br>CAMK inhibitor;<br>CDK inhibitor	MAPK signaling;Tyrosine Kinase/Adaptors;Cell cycle/Checkpoint	MAPKK2, MAPKKK2;ABL1, Lyn, HCK, Src;Breakpoint cluster region protein(BCR);Bcr-Abl ;CAMK;CDK 2	[1]
1-C10	THONZONIUM BROMIDE	C32H55BrN4O	591.71	ATPase inhibitor	others	(Na,K)-ATPase	
2-A7	AICAR	C9H14N4O5	258.24	AMPK activator	PI3K/Akt/mTOR Signaling	AMPK	[2]
2-C11	AT13148	C17H16ClN3O	313.78	Akt inhibitor	PI3K/Akt/mTOR Signaling	PKA;ROCK2;ROCK1;p70S6K ;Akt1;Akt3;SGK3;RSK1;Akt2	[3]
2-D11	CEP-33779	C24H26N6O2S	462.57	JAK inhibitor	JAK/STAT Singnaling	JAK2	[4]
3-A4	Ilaprazole sodium	C19H18N4O2S	366.44	proton pump inhibitor	Membrane Transporter/Ion Channel	proton pump	[5]
3-A7	OTSSP167	C25H28Cl2N4O2	487.42	MELK inhibitor	PI3K/Akt/mTOR Signaling	MELK	[6]

3-H3	Reversine	C21H27N7O	393.49	Adenosine receptor antagonist;Aurora kinase inhibitor	GPCR/G protein	human A3 adenosine receptor; Aurora A/B/C	[7]
3-E7	Caspofungin	C56H96N10O19	1213.42	Antifungal	Microbiology & Virology	1, 3-beta-D-glucan synthesis	[8]
3-F9	WH-4-023	C32H36N6O4	568.67	Src inhibitor	Angiogenesis	Src/Lck	[9]
4-E6	XAV939	C14H11F3N2OS	312.31	Wnt/beta-catenin inhibitor	Stem Cell	TNKS1/2	[10]
4-F6	CI994	C15H15N3O2	269.30	HDAC inhibitor	Chromatin/Epigenetics	HDAC1	[11]
4-H9	Afuresertib	C18H17Cl2FN4OS	427.32	Akt inhibitor	PI3K/Akt/mTOR Signaling	Akt1/2/3	[12]

Supplementary References

1. Remsing Rix LL, Rix U, Colinge J, *et al.* Global target profile of the kinase inhibitor bosutinib in primary chronic myeloid leukemia cells. Leukemia 2009;23(3):477-85.
2. Corton JM, Gillespie JG, Hawley SA, *et al.* 5-aminoimidazole-4-carboxamide ribonucleoside. A specific method for activating AMP-activated protein kinase in intact cells? Eur J Biochem 1995;229(2):558-65.
3. Yap TA, Walton MI, Grimshaw KM, *et al.* AT13148 is a novel, oral multi-AGC kinase inhibitor with potent pharmacodynamic and antitumor activity. Clin Cancer Res 2012;18(14):3912-23.
4. Seavey MM, Lu LD, Stump KL, *et al.* Therapeutic efficacy of CEP-33779, a novel selective JAK2 inhibitor, in a mouse model of colitis-induced colorectal cancer. Mol Cancer Ther 2012;11(4):984-93.
5. Seo KA, Lee SJ, Kim KB, *et al.* Ilaprazole, a new proton pump inhibitor, is primarily metabolized to ilaprazole sulfone by CYP3A4 and 3A5. Xenobiotica 2012;42(3):278-84.
6. Chung S, Suzuki H, Miyamoto T, *et al.* Development of an orally-administrative MELK-targeting inhibitor that suppresses the growth of various types of human cancer. Oncotarget 2012;3(12):1629-40.
7. D'Alise AM, Amabile G, Iovino M, *et al.* Reversine, a novel Aurora kinases inhibitor, inhibits colony formation of human acute myeloid leukemia cells. Mol Cancer Ther 2008;7(5):1140-9.
8. McGee WT, Tereso GJ. Successful treatment of Candida krusei infection with caspofungin acetate: a new antifungal agent. Crit Care Med 2003;31(5):1577-8.
9. Martin MW, Newcomb J, Nunes JJ, *et al.* Novel 2-aminopyrimidine carbamates as potent and orally active inhibitors of Lck: synthesis, SAR, and in vivo antiinflammatory activity. J Med Chem 2006;49(16):4981-91.
10. Dregalla RC, Zhou J, Idate RR, *et al.* Regulatory roles of tankyrase 1 at telomeres and in DNA repair: suppression of T-SCE and stabilization of DNA-PKcs. Aging (Albany NY) 2010;2(10):691-708.
11. Methot JL, Chakravarty PK, Chenard M, *et al.* Exploration of the internal cavity of histone deacetylase (HDAC) with selective HDAC1/HDAC2 inhibitors (SHI-1:2). Bioorg Med Chem Lett 2008;18(3):973-8.
12. Spencer A, Yoon SS, Harrison SJ, *et al.* The novel AKT inhibitor afuresertib shows favorable safety, pharmacokinetics, and clinical activity in multiple myeloma. Blood 2014;124(14):2190-5.

Supplementary Figure legends

Supplementary Figure 1. IPA-3 and auranofin potentiates colony formation inhibition in H23 and H520 cell lines.

(A, D) H23 (A) and H520 (D) cell colonies grown under single or combination treatment (IPA-3 and auranofin). Fixed colonies were stained using crystal violet.

(B, E) Concentrations of crystal violet were shown as ratio to control (non-treatment colonies defined as 1) in H23 (B) and H520 (E). Crystal violet was absorbed using 2% sodium dodecyl sulfate and measured at 570 nm.

(C, F) Combination index by each concentration of IPA-3 and auranofin. The lowest combination index in H23 (C) and H520 (F) was included synergism area (<0.9).

Supplementary Figure 2. Western blotting by treatment using single reagent or combination of IPA-3 plus auranofin in H23 and H520 cell lines. IPA-3 plus auranofin abrogated the expression and activation of protein signaling pathways involved in lung cancer in H23 and H520 cell lines.

H23 (A) and H520 (B) cell lines were exposed to 50 µM IPA-3, 5 or 10 µM auranofin (in H23 and H520 cell lines, respectively) or 10 µM trametinib and the combinations of IPA-3 plus auranofin or trametinib for 6 hours. Protein expression and activation were analyzed by Western blotting. Actin was used as house-keeping protein. The experiments were made at least twice.

Abbreviations: S, serine; T, threonine; Y, tyrosine

Supplementary Figure 3 Optimization and validation of the kinase assay for PAK1 inhibitor screening.

(A) The PAK1 kinase titration was performed using gradient concentrations of PAK1 with 100 µM ATP concentration for the indicated reaction time. The optimal PAK1 concentration is 7.5 nM and reaction time is 30 minutes.

(B) Gradient concentrations of ATP (0.1 µM to 2000 µM) were incubated with 7.5 nM PAK1 for 30 minutes and PAK1 kinase activity was measured.

(C) ATP Km (app) was calculated at 9.5 µM by fitting the data with Michaelis-Menten equation. Data are means ± SD (n = 3).

Supplementary Figure 4 Summary of cell growth inhibition using potential PAK1 inhibitors.

Based on the preliminary screening, 13 compounds showing more than 10% inhibition of cell viability at 10 μM were selected. The inhibition ratio, drug name and target of each 13 compound and were shown in table. IPA-3 was utilized as positive control.

Supplementary Figure 5 OTSSP167 plus auranofin potentiates colony formation inhibition in H23 and H520 cell lines

(A, D) H23 (A) and H520 (D) cell colonies grown under single or combination treatment (OTSSP167 and auranofin) were stained using crystal violet.

(B, E) Concentrations of crystal violet were shown as ratio to control (non-treatment colonies defined as 1) in H23 (B) and H520 (E). Crystal violet was absorbed using 2% sodium dodecyl sulfate and measured at 570 nm.

(C, F) Combination index by each concentration of OTSSP167 and auranofin. The lowest combination index in H23 (C) and H520 (F) was included synergism area (<0.9).

Supplementary Figure 6 Anti-tumor effects of single reagent or combination treatment using IPA-3, auranofin, and OTSSP167 in nude xenograft model in H23 and H520 cell lines.

(A, E) H23 (A) and H520 (E) cells were used for the xenograft model. Mice were treated once daily with the PAK1 inhibitor (IPA-3 or OTSSP167) single agent or combined with auranofin at given concentrations. Representative images of tumors measured at the end of the study compared with the control group are shown.

(B, F) Line graphs of tumor volumes of H23 (B) and H520 (F) after treatment by each therapeutic course (vehicle, single reagent, or combination). Tumor volumes were recorded every 2 days until 14th or 26th day. Errors bars mean +- SD of 5 animals per group. P values by comparing tumor volumes are described in the table.

(C, G) Histographs of tumor weight of H23 (C) and H520 (G) after treatment by each therapeutic course (vehicle, single reagent, or combination) at the time of sacrifice. P values by comparing tumor weight are described in the table.

(D, H) Line graphs of mice weight of H23 (D) and H520 (H) on every 2 days of each treatment course (vehicle, single reagent, or combination). P values by comparing mice body weight are described in the table.

Abbreviations: ig, orally intake (intragastric); ip, intraperitoneal injection

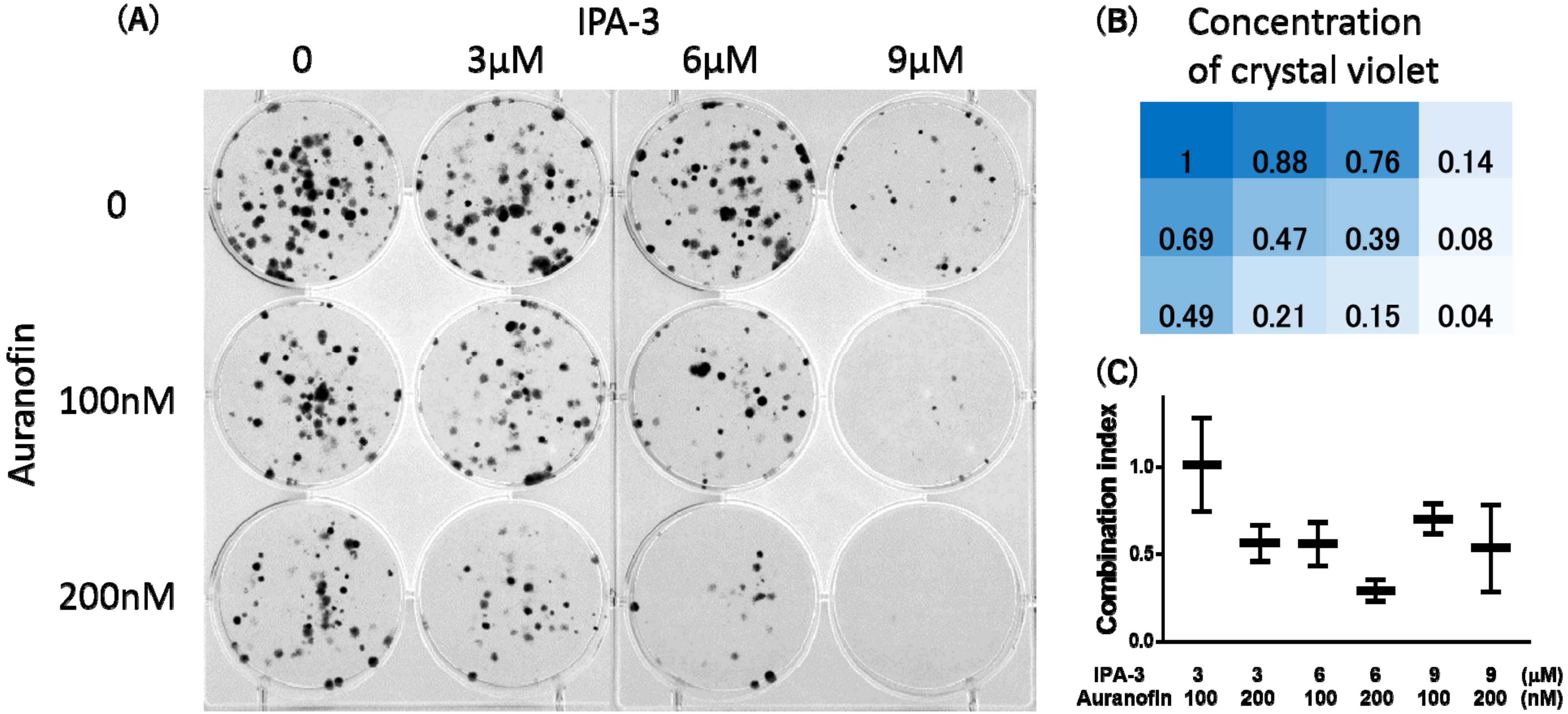

Supplementary Figure 1

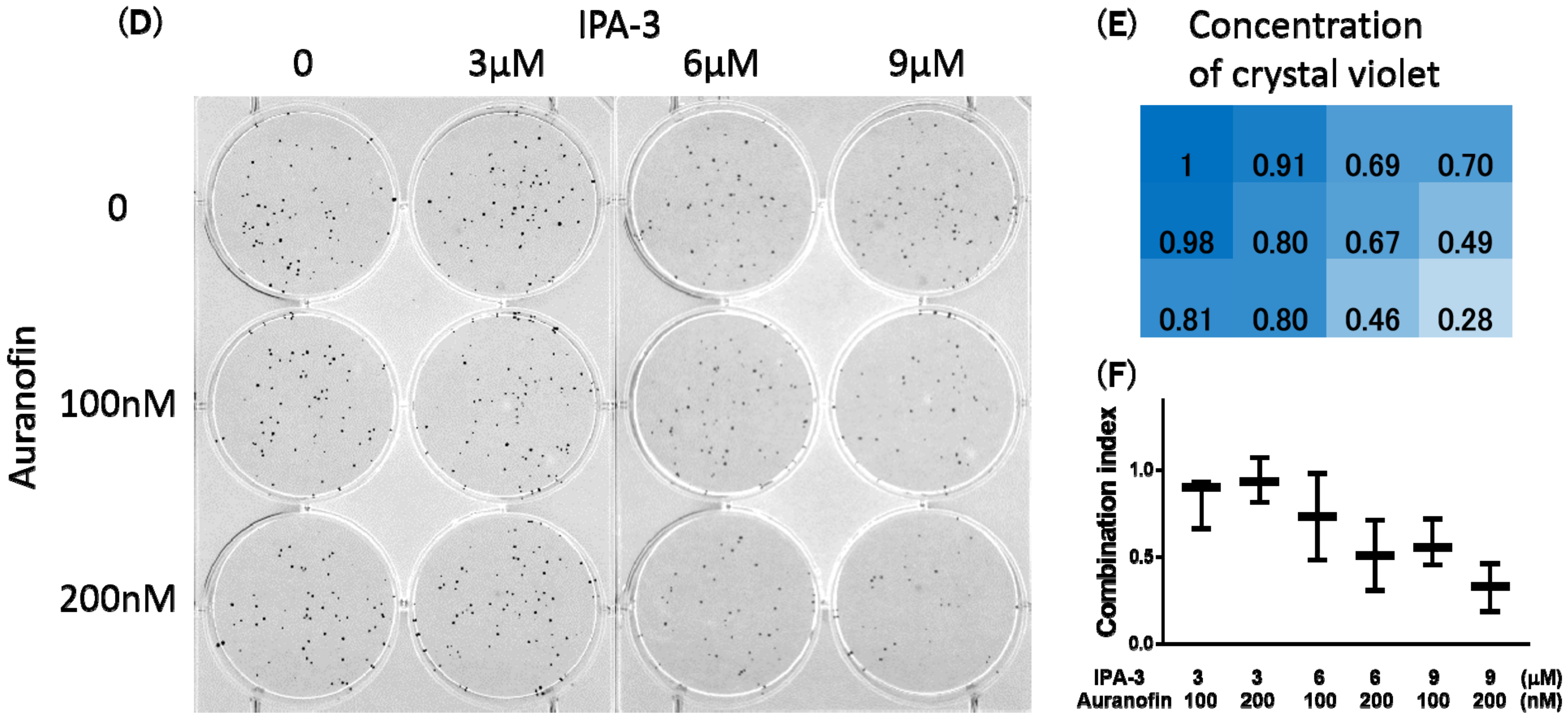

Supplementary Figure 1

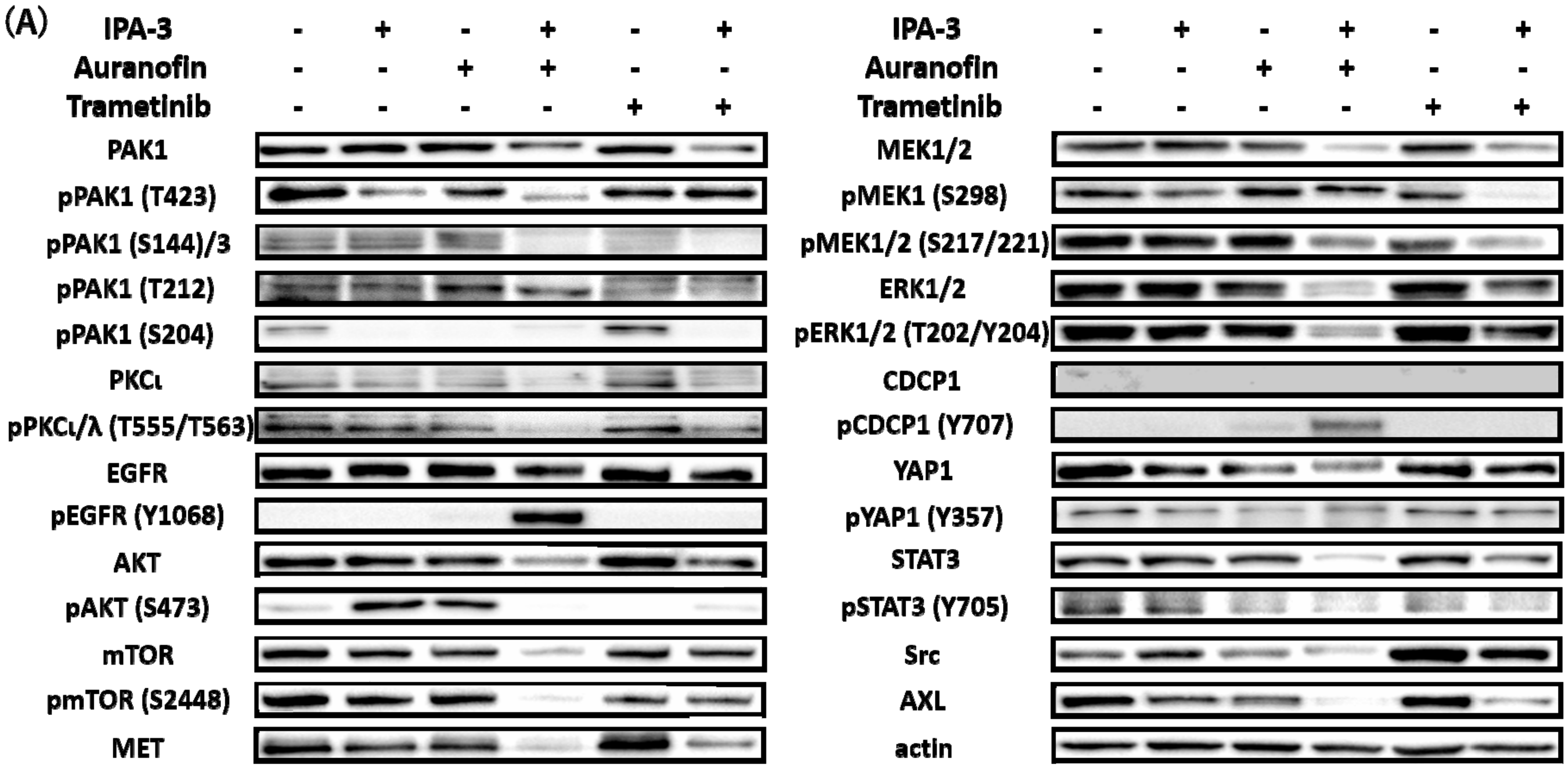

Supplementary Figure 2

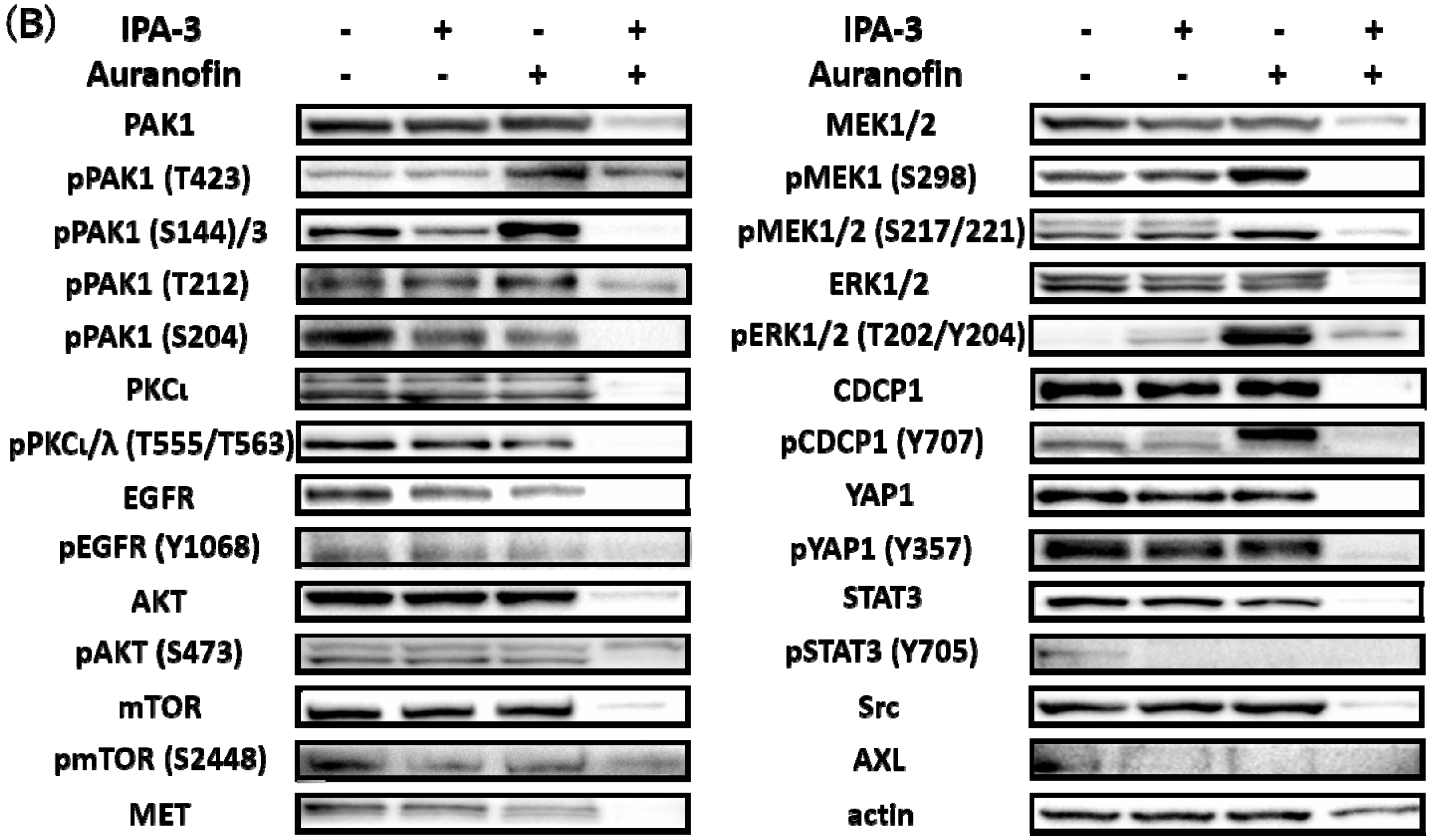

Supplementary Figure 2

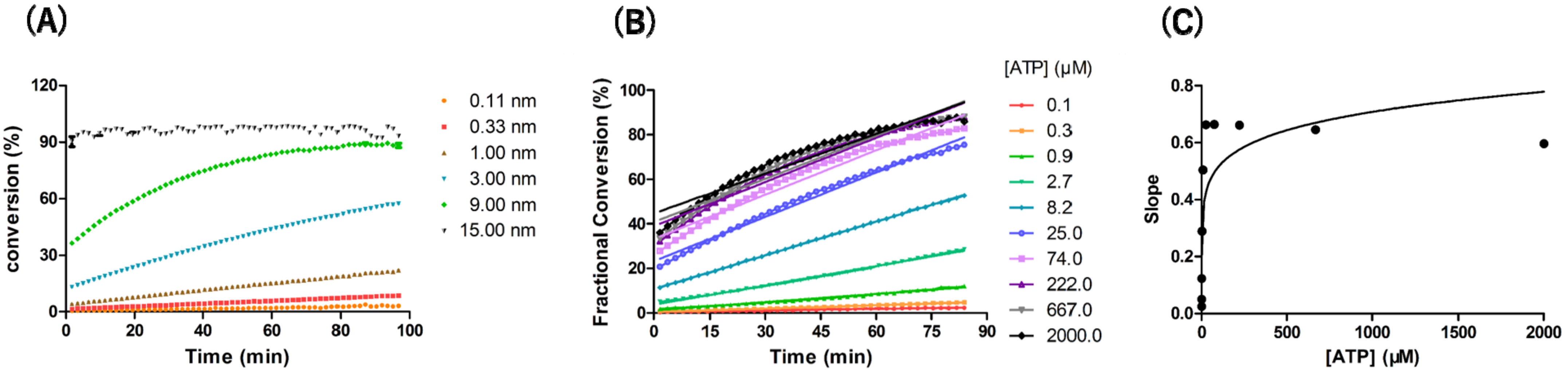

Supplementary Figure 3

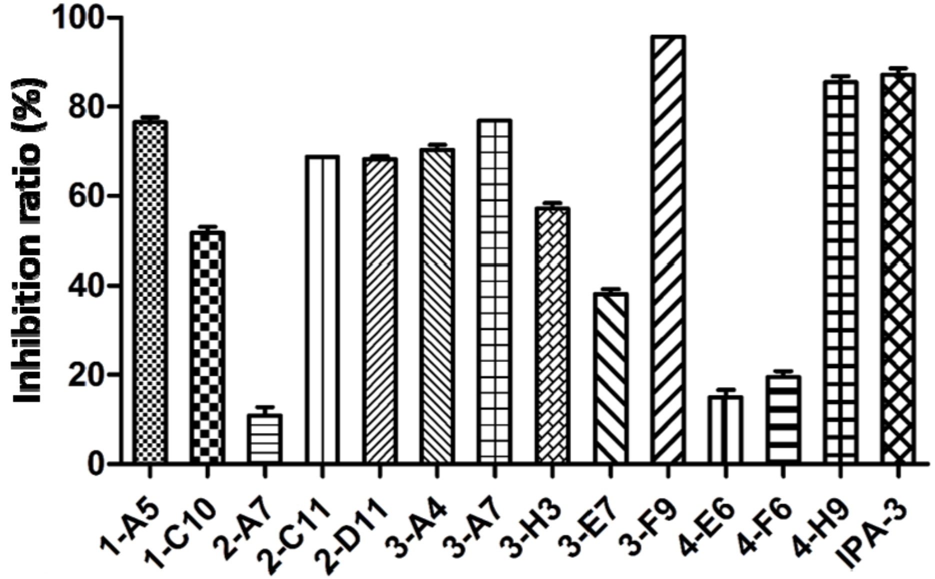

Symbol	Drug name	Target of inhibition
1-A5	Bosutinib	Tyrosine kinase
1-C10	Thonzonium bromide	(Na, K)-ATPase
2-A7	AICAR	*1
2-C11	AT13148	AGC kinase
2-D11	CEP33779	JAK2 kinase
3-A4	Ilaprazole sodium	Proton pump
3-A7	OTSSP167	MELK
3-H3	Reversine	Aurora A/B/C
3-E7	Caspofungin	Antifungal/Anti tumor
3-F9	WH-4-023	Lck/Src
4-E6	XAV939	Tankyrase 1/2
4-F6	CI994	HDAC/HDCA1
4-H9	Afuresertib	AKT

*1: activator of AMP-activated protein kinase

Supplementary Figure 4

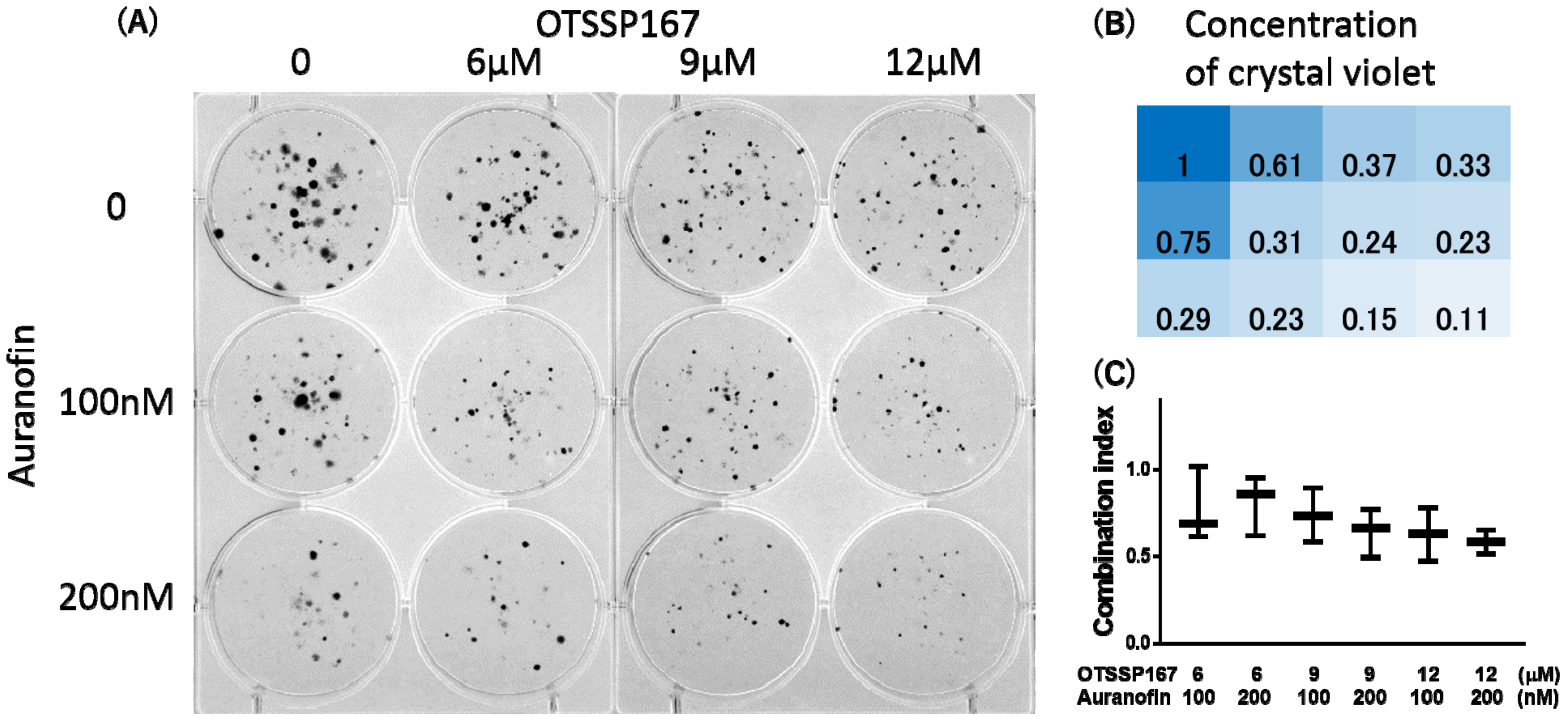

Supplementary Figure 5

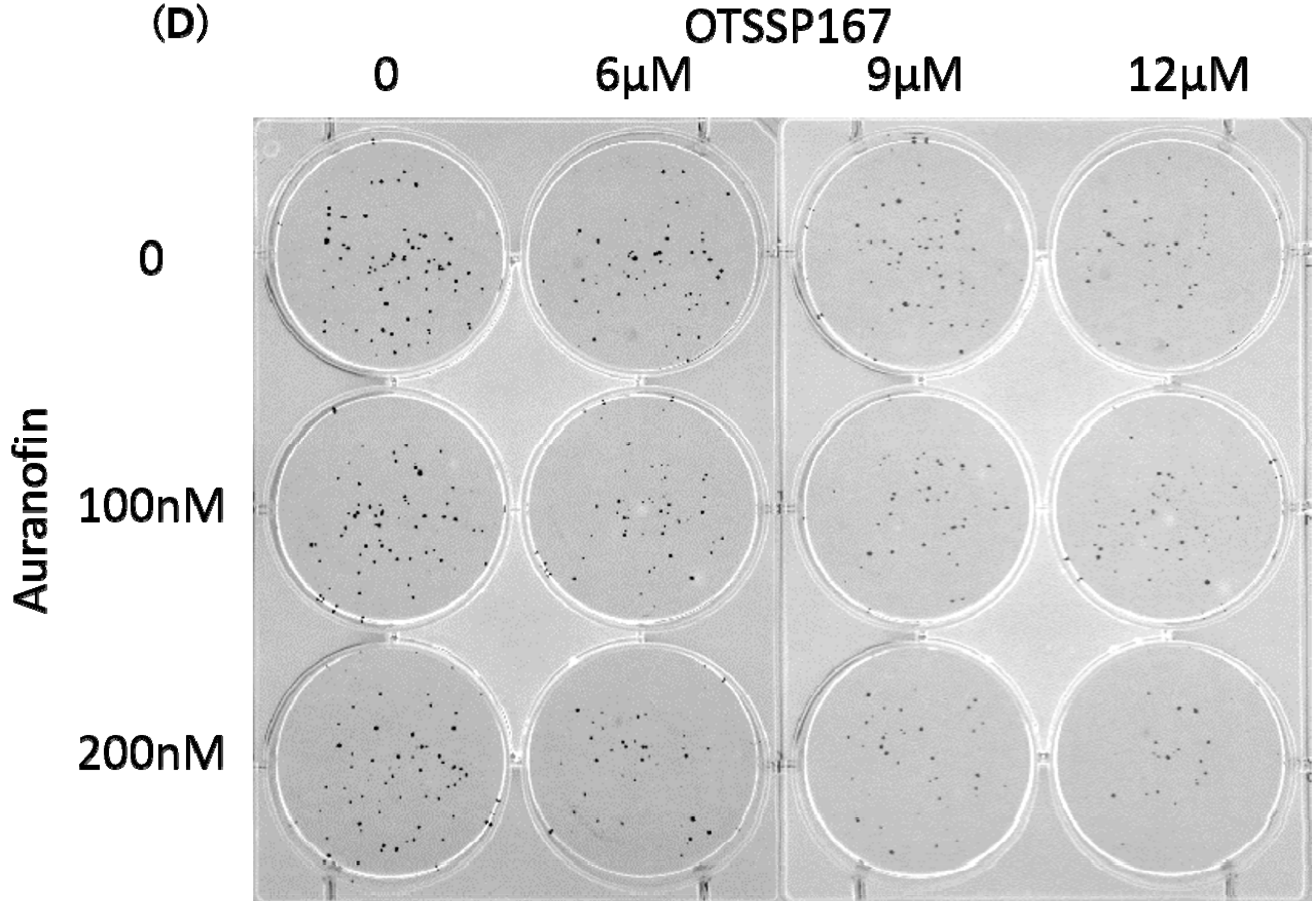

(E) Concentration of crystal violet

1	0.42	0.41	0.26
0.80	0.30	0.29	0.26
0.65	0.23	0.16	0.08

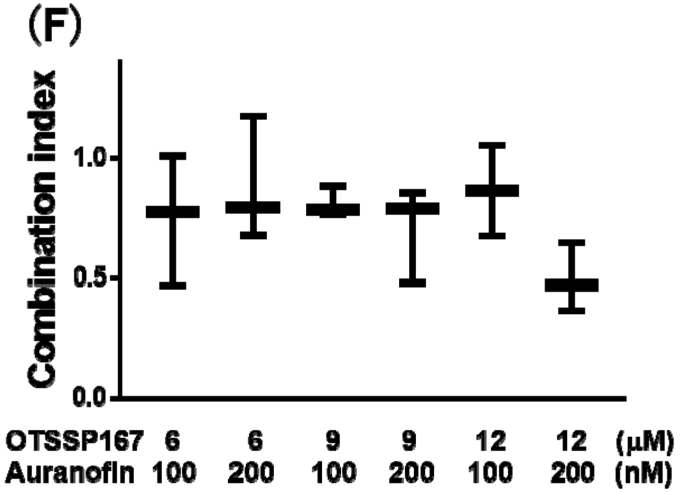

Supplementary Figure 5

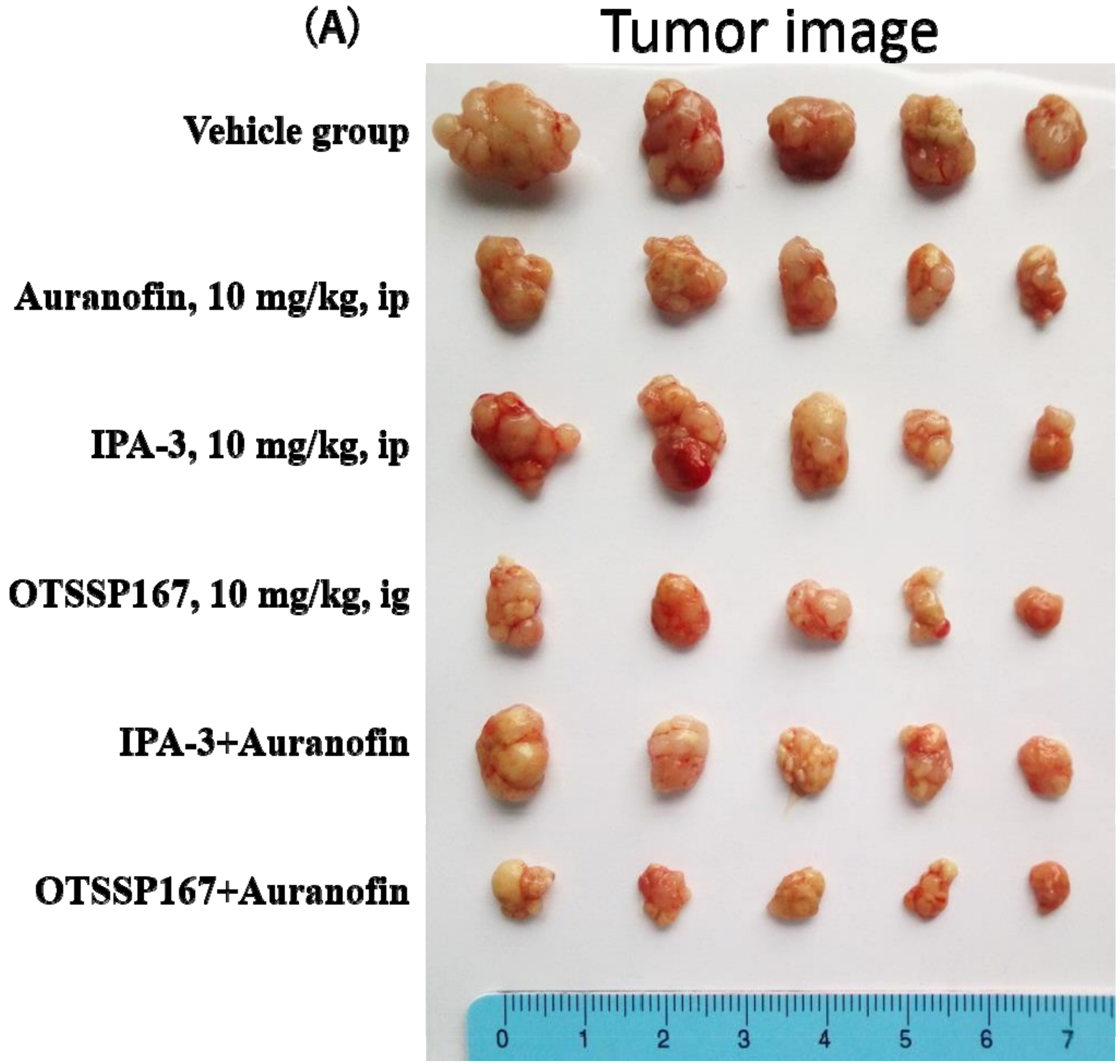

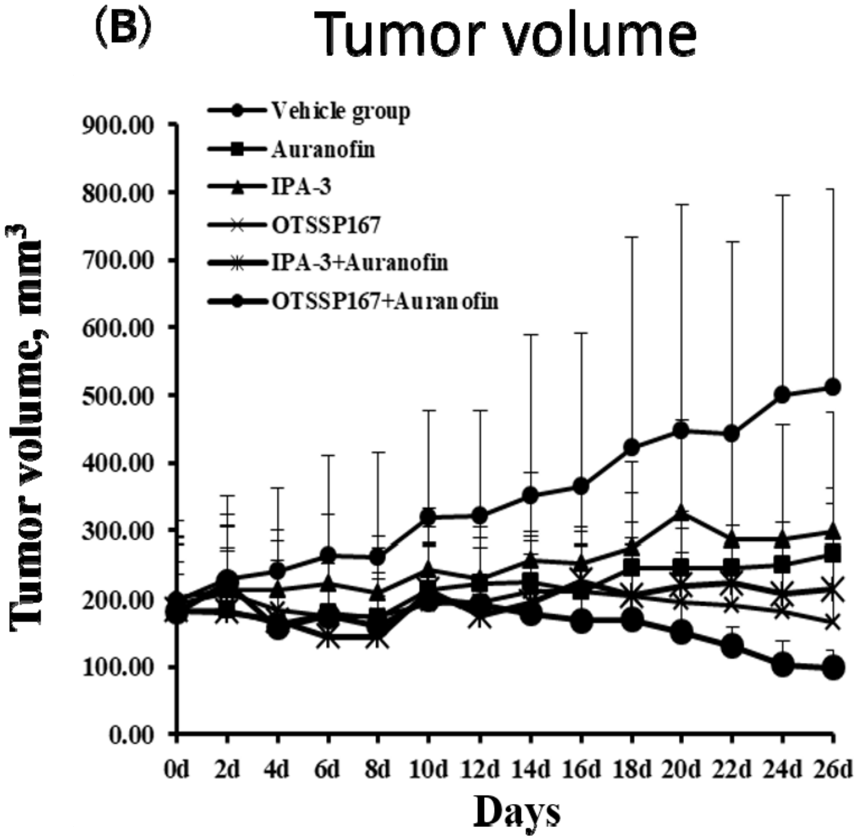

	IPA-3	Auranofin	OTSSP167
IPA-3 +Auranofin	0.408	0.483	
OTSSP167 +Auranofin		0.006	0.048

Supplementary Figure 6

(C)

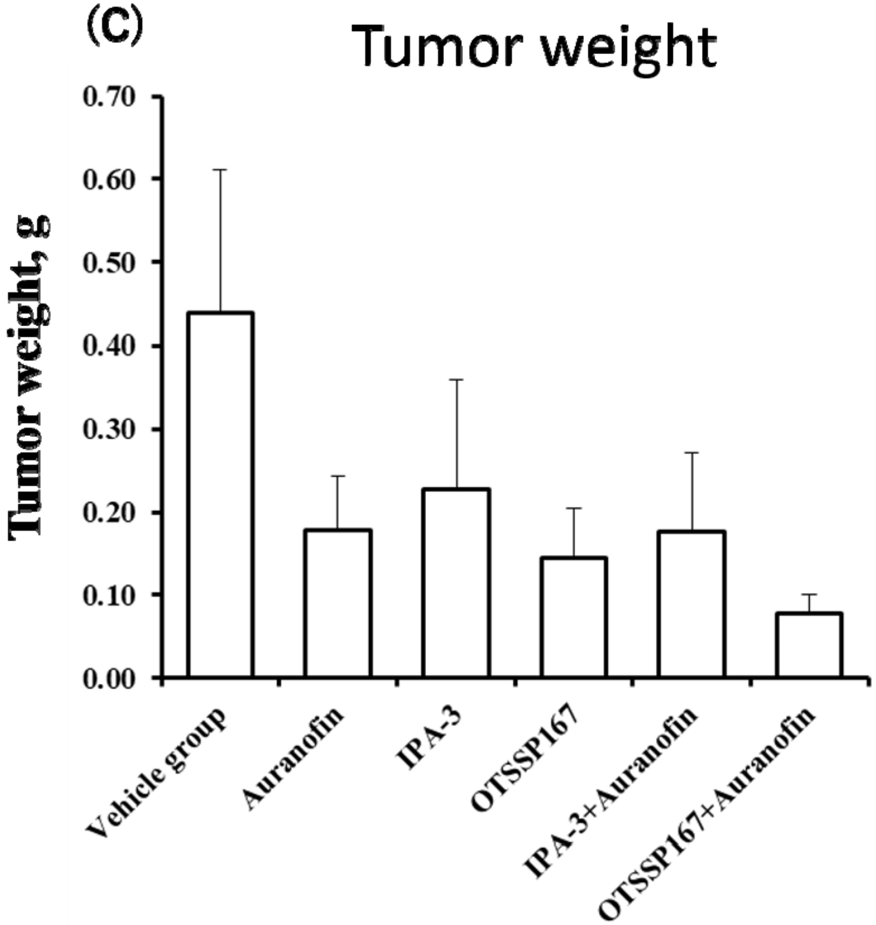

	IPA-3	Auranofin	OTSSP167
IPA-3 +Auranofin	0.497	0.975	
OTSSP167 +Auranofin		0.013	0.046

(D)

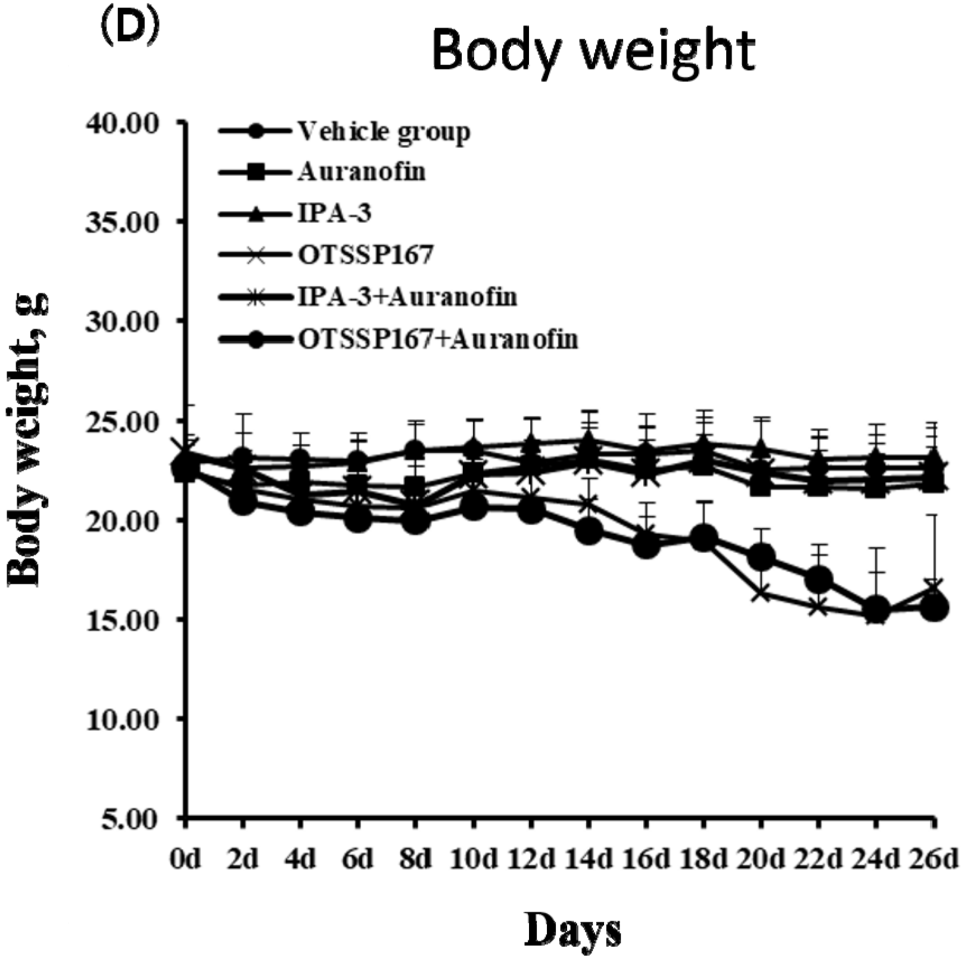

	IPA-3	Auranofin	OTSSP167	IPA-3 + Auranofin	OTSSP167 + Auranofin
vs Vehicle	0.601	0.301	0.002	0.606	<0.001

Supplementary Figure 6

(E) Tumor image

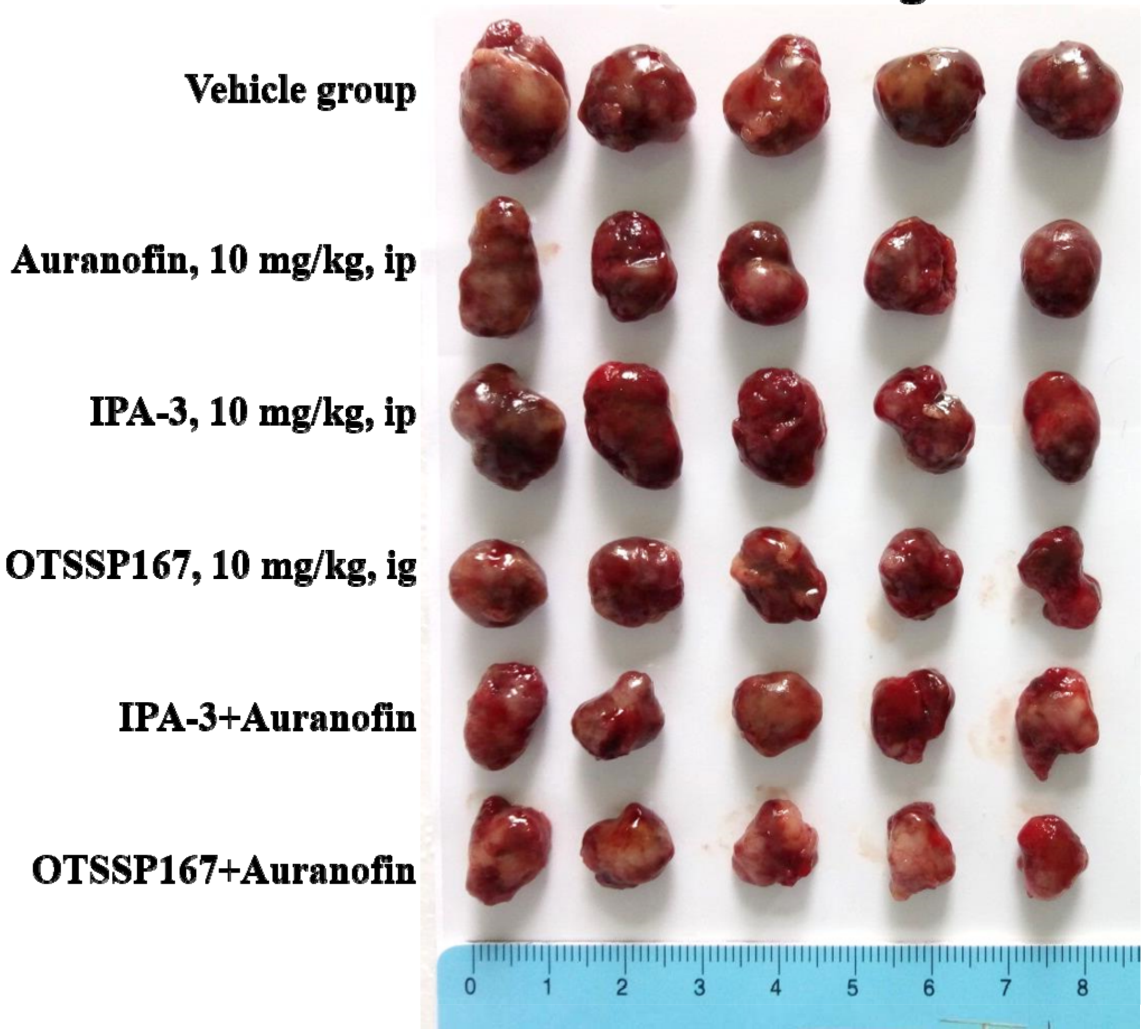

(F) Tumor volume

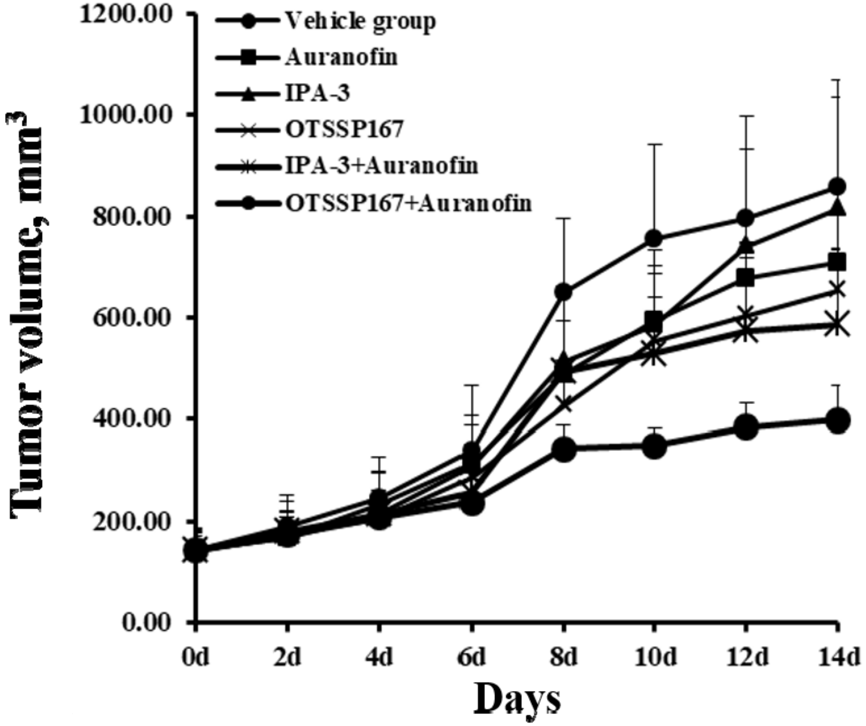

	IPA-3	Auranofin	OTSSP167
IPA-3 +Auranofin	0.059	0.079	
OTSSP167 +Auranofin		<0.001	<0.001

Supplementary Figure 6

(G)

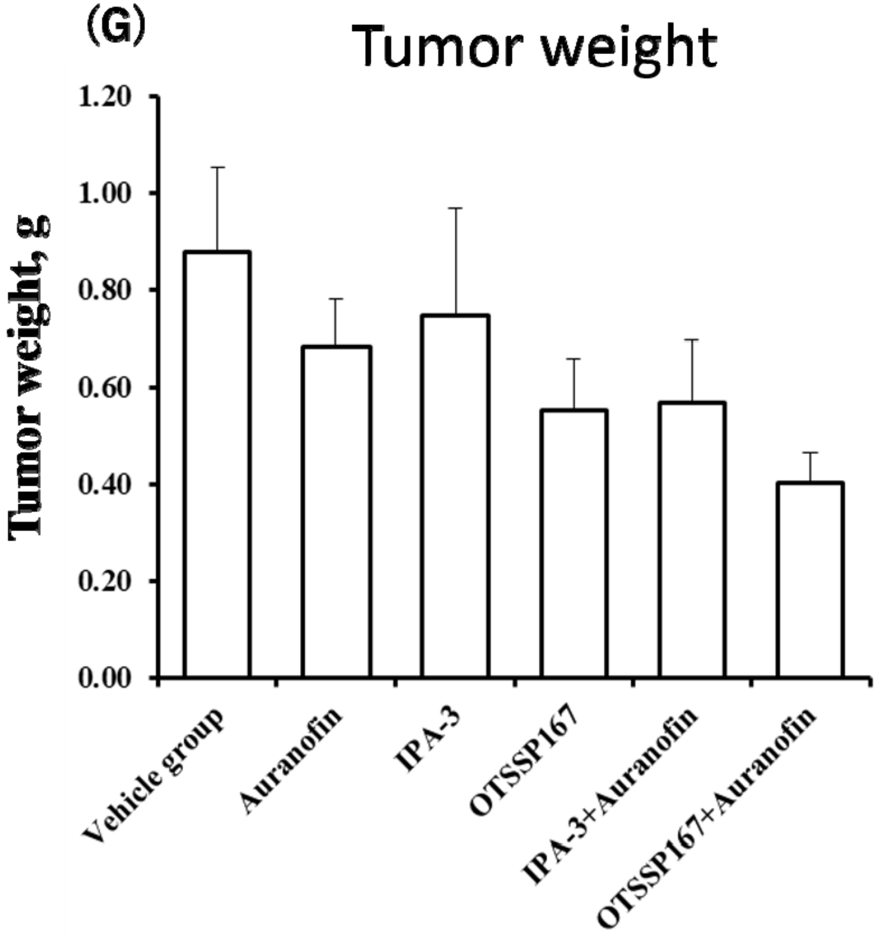

	IPA-3	Auranofin	OTSSP167
IPA-3 +Auranofin	0.065	0.059	
OTSSP167 +Auranofin		<0.001	0.004

(H)

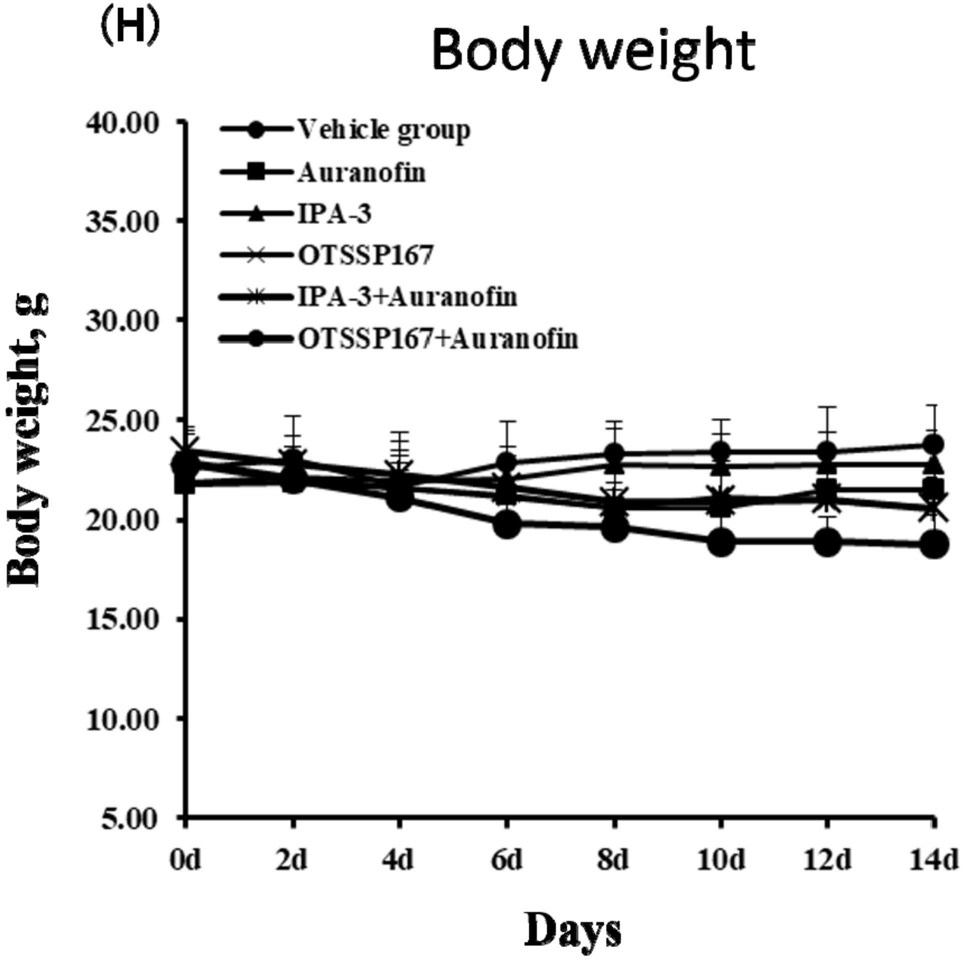

	IPA-3	Auranofin	OTSSP167	IPA-3 + Auranofin	OTSSP167 + Auranofin
vs Vehicle	0.324	0.196	0.008	0.004	<0.001

Supplementary Figure 6
